# Structural basis of PARP2 recognition of nucleosomal DNA breaks and inhibitor trapping

**DOI:** 10.1093/nar/gkag939

**Published:** 2026-10-05

**Authors:** Chathuni Jayathilake, Emily R Gregory-Lott, Rajbinder K Virk, Grace K Koh, Eun Cho, Tae Hun Kim

**Affiliations:** Department of Biochemistry, Case Western Reserve University, Cleveland 44106, OH, United States; Department of Biochemistry, Case Western Reserve University, Cleveland 44106, OH, United States; Department of Biochemistry, Case Western Reserve University, Cleveland 44106, OH, United States; Department of Biochemistry, Case Western Reserve University, Cleveland 44106, OH, United States; Department of Biochemistry, Case Western Reserve University, Cleveland 44106, OH, United States; Department of Biochemistry, Case Western Reserve University, Cleveland 44106, OH, United States; Case Comprehensive Cancer Center, Cleveland, OH, 44106, United States

## Abstract

Single-strand DNA breaks are among the most frequent forms of DNA damage, yet how PARP2 recognizes these lesions within the constrained geometry of chromatin remains unclear. Here we use cryogenic electron microscopy (cryo-EM) to determine structures of PARP2 and HPF1 bound to nucleosomes containing a site-specific single-strand break (SSB) at superhelical location + 5.3. The structures reveal a multivalent binding mode in which the Trp-Gly-Arg (WGR) domain engages the break while the helical domain and HPF1 contact the opposing DNA gyre, spanning both DNA gyres of the nucleosome. This configuration explains the higher affinity of PARP2 for nucleosomal SSBs relative to free DNA. 3D classification resolves multiple PARP2-bound states with distinct WGR orientations and DNA backbone distortions, revealing structural plasticity during damage recognition. Comparative analysis shows that HPF1 constrains the conformational ensemble of PARP2 and promotes a defined catalytic domain architecture compatible with histone PARylation. PARP inhibitors (olaparib, talazoparib, EB-47) stabilize WGR–helical domain interfaces and make the ADP-ribosyltransferase–HPF1 catalytic module dynamic, consistent with reverse allosteric retention on damaged chromatin, while also promoting asymmetric nucleosomal DNA unwrapping. Together, these structures provide a chromatin-context framework for understanding PARP2 recognition of nucleosomal DNA breaks and how PARPis reshape these interactions to promote PARP retention and local chromatin remodeling.

## Graphical Abstract

**Graphical Abstract:**
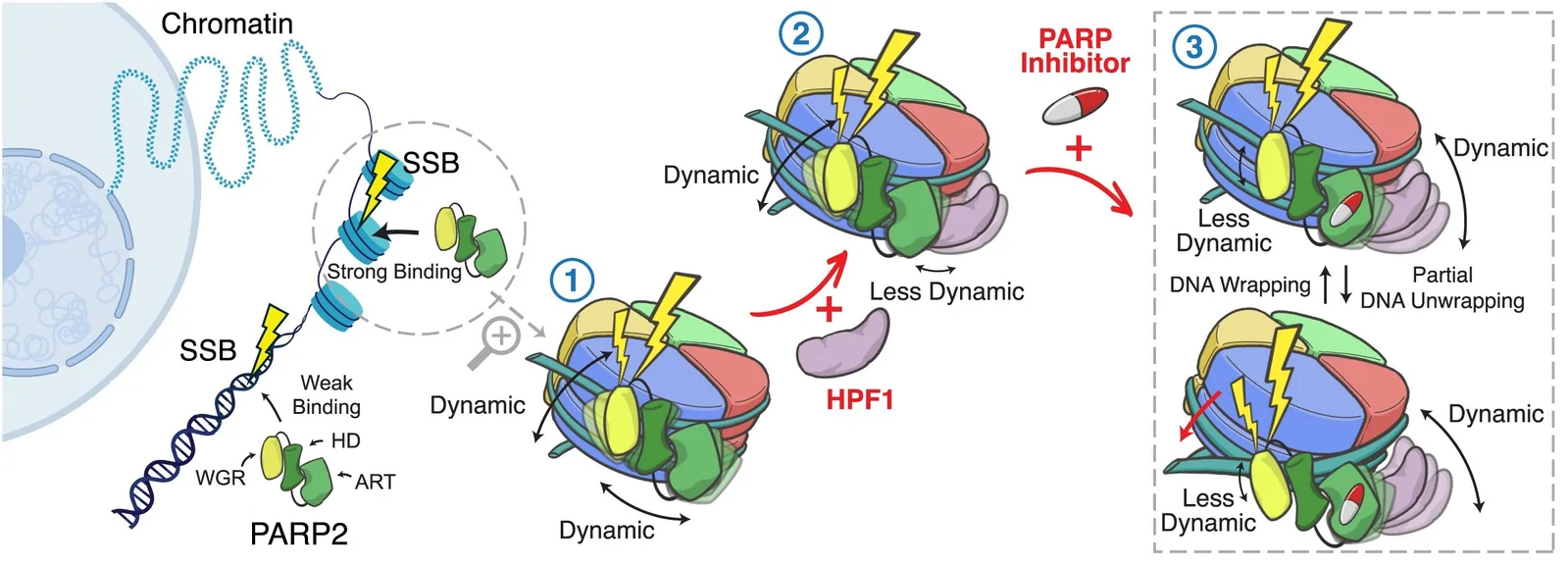

## Introduction

The stability of the genome is continuously threatened by DNA lesions arising from normal cellular metabolism, oxidative stress, and exogenous agents such as radiation and genotoxic drugs [1–4]. Among these lesions, single-strand breaks (SSBs) are particularly abundant, with tens of thousands estimated to arise in each mammalian cell per day [5–7]. Although often considered less deleterious than double-strand breaks (DSBs), unresolved SSBs can stall replication forks, collapse transcriptional machinery, and escalate into DSBs, thereby generating threats to cell viability and genome integrity [5, 6, 8]. Cells rely on base excision repair and related SSB repair pathways to counter this burden, rapidly detecting and processing SSBs before they interfere with essential chromosomal processes [6, 7, 9]. Importantly, many SSBs do not initially contain canonical 3′-hydroxyl and 5′-phosphate termini that can be directly sealed, as lesions generated by oxidative damage, abortive topoisomerase activity, or failed ligation can contain blocked or noncanonical ends that require end-processing enzymes such as PNKP, TDP1, or APTX before downstream gap filling and ligation can proceed [10–14]. Central to the broader detection and coordination of SSB repair are members of the poly(ADP-ribose) polymerase (PARP) family, including PARP1, PARP2, and PARP3, which act as DNA-damage sensors that bind sites of DNA damage, activate ADP-ribosylation, and recruit downstream DNA repair factors [1, 15–18].

The evolutionary distribution of these enzymes further underscores their specialized biological roles. PARP1-like clade 1 enzymes are broadly conserved across eukaryotes and are associated with an ancestral role in DNA damage response and genome integrity [19, 20]. PARP2 shows a more restricted phylogenetic profile than PARP1, though PARP2-clade members are not limited to vertebrates: PARP2 homologs are present across vertebrates and plants, with evidence for the PARP2 clade arising from an early eukaryotic gene duplication event [20]. In vertebrates, PARP1 and PARP2 have overlapping and non-redundant roles in maintaining genome stability [21, 22]. In plants such as *Arabidopsis thaliana*, a PARP2 ortholog bearing N-terminal SAP DNA-binding motifs contributes prominently to DNA damage and immune responses [23, 24]. PARP3/ARTD3 has been most extensively characterized in vertebrates, where it contributes to mitotic progression, DNA DSB repair, and chromatin-associated DNA break sensing [25–27].

Although these three DNA-dependent PARPs share a conserved C-terminal architecture composed of a tryptophan-glycine-arginine (WGR) domain followed by a catalytic (CAT) core, which is subdivided into helical (HD) and ADP-ribosyltransferase (ART) domains, their N-terminal architectures differ markedly [28, 29]. PARP1 is a large multidomain enzyme (∼116 kDa) that uses three N-terminal zinc-finger domains (Zn1, Zn2, and Zn3) for DNA damage recognition, together with a central automodification region containing a BRCT domain [28, 29]. By contrast, PARP2 (∼66 kDa) and PARP3 (∼60 kDa) lack these PARP1-specific zinc-finger and BRCT domains, instead containing short intrinsically disordered N-terminal regions upstream of their WGR domains [30]. In the context of chromatin, PARP1 and PARP2 interact with histone PARylation factor 1 (HPF1), which remodels the catalytic site to promote serine ADP-ribosylation on histones and other chromatin-associated proteins [31–35]. The clinical significance of PARP enzymes is underscored by the success of PARP inhibitors (PARPis), which exploit defects in homologous recombination repair to selectively kill BRCA1/2-mutant tumors, and which are now approved for ovarian, breast, pancreatic, and prostate cancers [36–42]. Beyond monotherapy, PARPis are being investigated in combination with radiotherapy and other chemotherapeutics, reflecting the central role of PARP enzymes in maintaining genome stability and their promise as therapeutic targets [43–49].

Given the central roles of PARP enzymes in DNA damage repair and their clinical importance as cancer therapeutic targets, particularly PARP1 and PARP2, extensive biochemical, structural, and biophysical studies have investigated how these enzymes recognize DNA breaks, undergo allosteric activation, and respond to PARPis. For PARP1, a crystallographic study using defined DNA substrates containing DSBs showed that the Zn1, Zn3, and WGR–CAT domains assemble as a monomeric complex on damaged DNA, forming a network of interdomain contacts that couples DNA break recognition to catalytic-domain destabilization [50]. NMR-based studies further showed that PARP1 recognizes SSBs through Zn1 and Zn2, with the WGR domain also contributing to DNA binding and assembly of the activated complex, and that HD-domain dynamics regulate substrate access, inhibitor effects, and allosteric activation [51, 52]. Complementary HDX-MS studies further revealed that DNA binding induces local unfolding of the autoinhibitory HD, thereby allowing productive NAD^+^ binding and stimulating PAR synthesis [53]. More recent cryo-EM and solution studies of full-length PARP1 bound to nicked DNA with HPF1 and Timeless showed that, even after assembly of the DNA-binding domains at the break, the catalytic region remains highly dynamic, supporting a model in which a tethered active catalytic module modifies substrates within a local radius around the lesion [54].

For PARP2, crystallographic studies showed that DNA damage recognition is mediated by the WGR domain, which binds 5′-phosphorylated DNA breaks, contacts DNA on both sides of the lesion, and can bridge DNA ends [55]. This 5′-phosphate dependence is important because cellular SSBs can contain diverse DNA termini and may require end processing before generating the canonical 3′-OH/5′-phosphate configuration recognized by downstream repair factors. Subsequent structures of PARP2 bound to DNA showed that this WGR-mediated binding is transmitted to the regulatory and catalytic domains, relieving autoinhibition and producing a catalytically competent state capable of binding NAD^+^ and HPF1 [56]. Cryo-EM studies conducted in a chromatin context further showed that PARP2–HPF1 can bridge nucleosome-proximal DNA breaks, stabilize HPF1 interaction, position the joint PARP2–HPF1 active site near histone H3 tails, and promote chromatin ADP-ribosylation [18, 57]. Recent HDX-MS work revealed an additional layer of PARP2-specific regulation, showing that PARP2 activation requires not only destabilization of the autoinhibitory HD but also melting of a PARP2-specific active-site helix, highlighting mechanistic differences between PARP1 and PARP2 that may be exploited for selective inhibitor design [58]. Despite these advances, most prior studies have examined PARP activation on free DNA or nucleosome-proximal DSB mimics, rather than a single 5′-phosphorylated SSB embedded within the nucleosome core. How PARP2 recognizes this constrained lesion geometry within nucleosomal DNA remains unresolved.

Here, we use cryo-EM to define how PARP2 engages DNA damage within the nucleosome. Structures of PARP2–HPF1 bound to nucleosomes containing an SSB at superhelical location (SHL) +5.3 reveal a multivalent binding mode in which the WGR domain recognizes the break while additional contacts with the two DNA gyres, corresponding to the superhelical turns of nucleosomal DNA wrapped around the histone octamer, stabilize the complex. 3D classification uncovers multiple PARP2 conformations that remodel nucleosomal DNA, highlighting the structural plasticity of PARP2 during damage recognition. Comparative analyses further show that HPF1 constrains the conformational ensemble of PARP2, allowing the stabilized ART domain to be resolved. In the presence of PARPis, the catalytic ART–HPF1 module becomes highly dynamic while DNA-binding interfaces remain stabilized, providing a structural framework for understanding how PARPis may alter PARP2 retention on damaged chromatin. In addition, inhibitor binding promotes partial unwrapping of nucleosomal DNA, revealing a potential mechanism by which PARPis alter local nucleosome structure at sites of DNA damage. Together, these structures establish a chromatin-context framework for understanding how PARP2 recognizes nucleosomal DNA breaks and how PARPis alter PARP2–nucleosome interactions.

## Materials and methods

### Polymerase chain reaction-based DNA production and purification

Widom 601 DNA templates containing a restriction enzyme recognition site (GTCTCN^N for Nt.BsmAI) at SHL +5.3 were designed to enable site-specific SSB cleavage. The template was synthesized by a commercial nucleic acid manufacturer (IDT) and supplied in lyophilized form. The 601 DNA sequence and the primers used are listed in Supplementary Table S1. The template DNA was resuspended in TE buffer (20 mM Tris, pH 8.0, 1 mM ethylenediaminetetraacetic acid (EDTA)] to a working concentration of 10 ng µl^−1^.

Primers flanking the 601 DNA sequence were designed with biotin modifications at the 5′ ends. Polymerase chain reaction (PCR) reactions were assembled on ice in a final volume of 100 µl containing 20 mM Tris–HCl (pH 8.8), 10 mM KCl, 10 mM (NH_4_)_2_SO_4_, 2 mM MgSO_4_, 0.1% (v/v) Triton X-100, 0.1 mg ml^−1^ nuclease-free bovine serum albumin, 0.8 mM dNTPs, 5 µM each forward and reverse primer, 42 nM Pfu-Sso7d DNA polymerase, 0.5 ng µl^−1^ template DNA, and molecular-biology-grade water [59]. PCR amplification was performed using the following cycling conditions: initial denaturation at 94°C for 5 min; 35 cycles of denaturation at 94°C for 30 s, annealing at 62°C for 30 s, and extension at 72°C for 1 min; followed by a final extension at 72°C for 2 min using a Bio-Rad T100 thermal cycler.

PCR products were purified using a DNA clean-up kit (QIAGEN) and subsequently used as templates for large-scale PCR amplification. Reaction volumes were scaled up to 10 ml and performed in 96-well plates. The amplified DNA was purified by anion-exchange chromatography using a Mono Q column (Pharmacia) operated on an ÄKTA chromatography system (Cytiva). The column was equilibrated in Buffer A (20 mM Tris–HCl, pH 8.0, 1 mM EDTA), and DNA was eluted using a linear NaCl gradient with Buffer B (20 mM Tris, 1 M NaCl, 1 mM EDTA, pH 8.0).

### Site-specific nicking of DNA using Nt.BsmAI-R386D

Purified DNA was buffer-exchanged into a no-NaCl buffer containing 50 mM Tris, 1 mM EDTA, pH 8.0 prior to nicking reactions. Site-specific nicking was performed using the strand-specific nicking endonuclease Nt.BsmAI-R386D [59]. Reactions contained 50 mM Tris, 250 mM NaCl, 10 mM MgCl_2_, pH 8.7, 500 nM DNA, and 100 nM Nt.BsmAI-R386D, and were incubated overnight at 37°C. Cleavage products were analyzed by polyacrylamide gel electrophoresis under both denaturing and native conditions. Denaturing PAGE was performed using 15% polyacrylamide gels containing 8 M urea, and native polyacrylamide gel electrophoresis (PAGE) was performed using 6% polyacrylamide gels. Gels were run at 150 V for 45 min (XCell SureLock™ Mini-Cell, Invitrogen). For denaturing PAGE, samples were heated to 95°C for 5 min prior to loading in the presence of loading buffer (1.3 × tris-borate-EDTA [TBE], 2.66 M urea, 8% w/v Ficoll PM400); running buffer (0.5× TBE) was preheated to 65°C. DNA was visualized by staining with Safe-Red DNA stain. Following confirmation of nicking, DNA was purified using a MonoQ anion-exchange column and used for nucleosome reconstitution.

### Histone octamer reconstitution and nucleosome assembly

Histone octamers were reconstituted from individual histones H2A, H2B, H3, and H4 at a molar ratio of 1.2:1.2:1:1. For fluorescent labeling, either H2A-Cys or H3-Q125C was labeled with Alexa Fluor™ 488 C5 Maleimide (Invitrogen) via maleimide–thiol chemistry. For octamer reconstitution, histones were dissolved in unfolding buffer (25 mM Tris, pH 7.5, 6 M guanidine–HCl) and assembled into octamers by stepwise dialysis against assembly buffer (10 mM Tris, pH 7.4, 2 M NaCl, 1 mM EDTA) at 4°C using a GeBaFlex dialysis cassette (GeBA). When fluorescent labeling was performed, 2 mM of dithiothreitol (DTT) was included in the dialysis and unfolding buffers. The final octamer concentration was aimed for 40–70 µM. Assembled octamers were purified by size-exclusion chromatography on a Superdex 200 10/300 column (Cytiva), verified by sodium dodecyl sulphate–polyacrylamide gel electrophoresis (SDS–PAGE) run at 200 V for 30 min, and quantified using a NanoDrop spectrophotometer.

For nucleosome reconstitution, nucleosomal DNA containing an SSB at SHL +5.3 and purified histone octamer were buffer-exchanged into RB High buffer (10 mM Tris, pH 7.4, 2 M KCl, 1 mM EDTA and 2 mM DTT for fluorescent samples). DNA and histone octamers were combined at a 1:1 molar ratio to reach a final concentration of 6 µM each. The mixture was subjected to gradient dialysis at 4°C using a dialysis cassette submerged in 200 ml RB High buffer into RB Low buffer (10 mM Tris–HCl, 150 mM KCl, 1 mM EDTA and 2 mM DTT for fluorescent samples) by dripping 1 l of pre-chilled RB Low buffer into the beaker containing the cassette at 1 ml min^−1^ under continuous stirring at 4°C. Nucleosome assembly was verified on a 6% native PAGE run at 150 V for 50 min. The running buffer was pre-chilled, and gels were maintained on ice or in a cold room during electrophoresis. Nucleosomes were visualized by fluorescence (Alexa Fluor 488) and Safe-Red staining. Samples were cleared of aggregates by centrifugation at 13 000 rpm for 5 min at 4°C. Concentrations were determined by measuring DNA absorbance at 260 nm using a spectrophotometer, and samples were stored in RB Low buffer at 4°C.

### Protein expression and purification

#### PARP2

The amino acid sequence of human PARP2 isoform 1 (UniProt ID: Q9UGN5-1) was used as the reference protein sequence for designing all PARP2 expression constructs used in this study. PARP2 construct (residues 90–583) lacking the first 89 amino acids and PARP2-WGR construct (residues 90–212) were generated by PCR and cloned into the pET-SUMO expression vector using Gibson assembly. The resulting plasmids were transformed into *Escherichia coli* (*E. coli*) Rosetta 2 pLysS cells for protein expression. Large-scale cultures were grown at 37°C in Luria-Bertani (LB) medium supplemented with 50 µg ml^−1^ kanamycin, 100 µg ml^−1^ chloramphenicol, and 1.2 mg ml^−1^ benzamide until an OD_600_ of ∼0.7 was reached. Cultures were then shifted to 20°C and induced with 0.5 mM isopropyl β-D-1-thiogalactopyranoside (IPTG) for overnight expression. Cells were harvested and lysed in the presence of a protease inhibitor cocktail. The clarified lysate was applied to a Ni-NTA affinity column equilibrated with binding buffer (25 mM Tris, 500 mM NaCl, 20 mM imidazole, 2 mM β-mercaptoethanol, pH 8.0). After washing with ∼20 column volumes of binding buffer, proteins were eluted with elution buffer (25 mM Tris, 500 mM NaCl, 300 mM imidazole, 2 mM β-mercaptoethanol, pH 8.0) and treated with ULP1 protease to cleave 6xHis-SUMO during dialysis against dialysis buffer (25 mM Tris, 150 mM NaCl, 2 mM β-mercaptoethanol, 5% glycerol, pH 8.0). Protease-digested samples were reapplied to a second Ni-NTA column in the presence of 30 mM imidazole to remove affinity tags. The flow-through was further purified by HiTrap Heparin chromatography using buffer A (25 mM Tris, 350 mM NaCl, 2 mM β-mercaptoethanol, 5% glycerol, pH 8.0) and buffer B (25 mM Tris, 1 M NaCl, 2 mM β-mercaptoethanol, 5% glycerol, pH 8.0), followed by size-exclusion chromatography on a Superdex 200 10/300 column (Cytiva) equilibrated using SEC buffer (25 mM Tris, 150 mM NaCl, 2 mM β-mercaptoethanol, pH 8.0). Purified proteins were flash-frozen in liquid nitrogen and stored at −80°C in the presence of 10% (v/v) glycerol.

#### HPF1

The human HPF1 (UniProt ID: Q9NWY4) expression plasmid (pET30-HPF1-His-SUMO-Flag, Addgene #111577) was obtained from Addgene. Protein was expressed in *E. coli* BL21(DE3)-CodonPlus (RIPL) cells. Large-scale cultures were grown at 37°C in LB medium supplemented with 50 µg ml^−1^ kanamycin and 100 µg ml^−1^ chloramphenicol until an OD_600_ of ∼0.7 was reached, then shifted to 16°C and induced with 0.5 mM IPTG for overnight expression. Ni-NTA affinity column, ULP1 cleavage, and a second Ni-NTA column were performed as described above. The second Ni-NTA flow-through was further purified using an SP ion-exchange column followed by Superdex 200 size-exclusion chromatography. Purified proteins were flash-frozen in liquid nitrogen and stored at −80°C in the presence of 10% (v/v) glycerol.

### Histone preparation

Recombinant histones H2A, H2B, H3, and H4 from *Drosophila melanogaster* were produced in *E. coli* BL21(DE3)-CodonPlus (RIPL) using established expression and purification strategies [32, 60]. Coding sequences were cloned into pET21b vectors. Histone H2B was engineered with an N-terminal hexahistidine tag followed by a tobacco etch virus (TEV) protease cleavage site, whereas H2A, H3, and H4 were expressed without affinity tags. To prevent aberrant disulfide bond formation, histone H3 contained a C110S substitution. Additionally, an extra isoleucine residue was introduced immediately following the initiator methionine of H4 and downstream of the TEV cleavage site in H2B to enhance expression yield. For site-specific fluorescent labeling, an additional cysteine residue was introduced at the C-terminus of H2A, and Q125 of H3 was mutated to cysteine.

Proteins were expressed in *E. coli* BL21(DE3)-CodonPlus (RIPL) cells grown in LB medium at 37°C to mid-log phase (OD_600_ of 0.8). Expression was induced by adding 1 mM IPTG. Cultures were incubated overnight at 37°C following induction, except for H4, which was expressed for 5 h.

### Purification of untagged histones H2A, H3, and H4

Cell pellets were resuspended in wash buffer containing 50 mM Tris, 100 mM NaCl, pH 7.5, and 1% (v/v) Triton X-100, followed by lysis via sonication. Insoluble fractions containing histones were collected by centrifugation, washed once in detergent-free buffer (50 mM Tris, 100 mM NaCl, pH 7.5), and subsequently solubilized in 6 M guanidine hydrochloride.

The denatured proteins were dialyzed into urea buffer consisting of 7 M urea, 50 mM sodium phosphate, 50 mM NaCl, and 1 mM EDTA (pH 7.4). Urea solutions were pretreated with Amberlite resin to limit carbamylation. Histones were then purified by cation-exchange chromatography using SP resin with a linear NaCl gradient ranging from 50 mM to 1 M. Fractions containing the desired histones were pooled, dialyzed extensively against water, lyophilized (Labconco FreeZone 2.5 l −50°C), and stored at −20°C.

### Purification of His-tagged H2B

Cell pellets expressing H2B were solubilized in denaturing Ni-NTA binding buffer comprising 6 M guanidine hydrochloride, 20 mM sodium phosphate, 500 mM NaCl, and 20 mM imidazole (pH 7.5). Clarified lysates were applied to Ni-NTA resin, washed extensively using binding buffer, and the bound protein was eluted with buffer containing 300 mM imidazole.

For removal of the affinity tag, the eluted H2B was rapidly diluted into TEV protease cleavage buffer (20 mM sodium phosphate, 500 mM arginine hydrochloride, 2 mM DTT, pH 7.4) and dialyzed overnight at 4°C against dialysis buffer (25 mM Tris, 150 mM NaCl, 2 mM DTT, pH 7.4) in the presence of TEV protease. The cleaved His tag and TEV protease were removed by a second passage over Ni-NTA resin performed in the presence of 30 mM imidazole. Finally, H2B samples were dialyzed into water, lyophilized, and stored at −20°C.

### ScFv preparation

The expression and purification of scFv were performed as described previously [61]. The TEV-scFv20 construct [61] was synthesized and subcloned into pET21b to generate a His-TEV-scFv expression construct. After transformation into *E. coli* Rosetta 2(DE3) pLysS, cells were grown to an OD_600_ of ∼0.8 and induced with 0.5 mM IPTG at 23°C overnight. Cells were harvested and resuspended in wash buffer (50 mM Tris, 1 mM EDTA, 100 mM NaCl, 1% (v/v) Triton X-100, pH 7.4) and lysed by sonication. After centrifugation, the soluble fraction was removed and the pellet was resuspended in wash buffer lacking Triton X-100, followed by a second centrifugation. The pellet was then solubilized in denaturation buffer (100 mM Tris, 6 M guanidine–HCl, 2 mM DTT, pH 7.4) and refolded by rapid dilution into refolding buffer (50 mM Tris, 0.5 M arginine-HCl, 2 mM GSH, 1.3 mM GSSG, 1 mM EDTA, pH 8.0). The refolded protein was dialyzed twice against dialysis buffer (25 mM Tris, 115 mM urea, pH 7.4) at 4°C. After removing precipitated material by centrifugation, the supernatant was loaded onto an SP column, and scFv was purified using a 0–1 M NaCl gradient. The eluted protein was further purified by size-exclusion chromatography on a HiLoad 16/600 Superdex 75 pg column equilibrated with gel-filtration buffer (20 mM Tris–HCl, 50 mM NaCl, 0.5 mM EDTA, 10% glycerol, pH 7.4). The purified scFv was stored at 4°C until use.

### Affinity measurements by electrophoretic mobility shift assay 

Electrophoretic mobility shift assay (EMSA) was performed to assess the binding of PARP2 to nucleosomes or free DNA containing an SSB. The target molecule (nucleosomes or DNA) was maintained at a constant concentration of 100 nM, while ligand protein concentrations were titrated at 0, 50, 100, 200, 300, and 400 nM. Binding reactions were assembled in a binding buffer containing 10 mM Tris, 30 mM KCl, 2 mM DTT, pH 7.5. For the nucleosome-PARP2 binding assay, if indicated, 5 µM HPF1 was included in the reaction mixture. To assess PARP2-HPF1 binding to SSB in the nucleosomes in the presence of scFv, an EMSA experiment was performed in the presence of 0.5 μM scFv. Samples were incubated at room temperature for 30 min to allow complex formation.

Reaction mixtures were resolved on 6% native polyacrylamide gels at 150 V for 80 min at 4°C. Gels were imaged using fluorescence detection to visualize Alexa Fluor 488-labeled nucleosomes, and Safe-Red staining was used to detect free DNA. Band intensities corresponding to bound and unbound species were quantified using FIJI. Reported values represent the mean ± standard deviation (SD). from *n* = 2 independent EMSA experiments. Binding curves were generated using Python3 and fit to the Hill equation to determine dissociation constants (*K*_d_).

### PARylation assays

PARylation reactions were carried out in 25 µl volumes containing 25 mM Tris, 112.5 mM NaCl, 37.5 mM KCl, 2 mM MgCl_2_, 2 mM DTT, pH 7.5. Reactions included an increasing concentrations (0, 50, 250, 500, 1000 µM) of NAD^+^, 200 nM histone H3-Alexa Fluor 488-labeled nucleosomes, 1 µM PARP2 (residues 90–583), and 3 µM HPF1. Samples were incubated at 37°C for 25 min, and the reaction was terminated by adding 4× SDS loading buffer. Reaction products were resolved by SDS–PAGE, and PARylation of histone H3 (A488) was detected by fluorescence imaging.

For PARylation assays performed in the presence of PARPis, inhibitor concentrations of 0, 0.5, 1.0, 1.5, and 3.0 µM were tested while all other reaction components were maintained constant as mentioned above. The concentration of NAD⁺ was held constant at 1 mM throughout all reactions. Following incubation, samples were resolved by SDS–PAGE, and PARylation of Alexa Fluor 488-labeled histone H3 (H3-A488) was analyzed by fluorescence imaging.

### Cryo-EM sample assembly and data acquisition

Prior to complex formation, all nucleosome and protein samples were exchanged into cryo-EM buffer composed of 10 mM Tris, 30 mM KCl, and 2 mM DTT, pH 7.5. Unless otherwise indicated, all concentrations listed below are final concentrations in the assembled vitrification sample after accounting for dilution upon addition of all components. Complexes were assembled using the same order of addition for apo and inhibitor-bound PARP2–HPF1 samples. First, nucleosomes containing an SSB at SHL +5.3 (2 µM) were incubated with scFv (8 µM) for 15 min at ambient temperature. Second, PARP2 (residues 90–583; 5 µM) and HPF1 (8 µM) were added to the nucleosome–scFv mixture. For inhibitor-bound samples, PARPis (EB-47, talazoparib, or olaparib; 10 µM) were included together with PARP2 and HPF1 at this addition step. For WGR-only samples, the isolated WGR domain (residues 90–212; 8 µM) was added instead of PARP2 and HPF1. PARPis were prepared as stocks in 100% DMSO and diluted into the sample to a final dimethyl sulfoxide (DMSO) concentration of 0.1% before vitrification. The resulting mixtures were incubated for an additional 25 min at room temperature to promote complex assembly. No chemical crosslinking, gel filtration, or other enrichment or purification steps were performed before vitrification.

Cryo-EM grids were prepared using Quantifoil R2/1 copper grids (300 mesh) with a carbon support layer. Grids were glow-discharged immediately prior to use using 15 mA, for 60 s. Aliquots (3 µl) of assembled complexes were applied to grids using a Vitrobot Mark IV (Thermo Fisher Scientific) operated at 4°C and 100% relative humidity. Excess liquid was removed by a single blotting step lasting 3–4 s (blot force 0, wait time 5 s), followed by rapid vitrification in liquid ethane. Prepared grids were stored in liquid nitrogen until imaging.

Cryo-EM data were collected on a Titan Krios G3i transmission electron microscope (Thermo Fisher Scientific) operated at 300 kV and equipped with an X-FEG electron source, a BioQuantum energy filter, and a K3 direct electron detector (Gatan). Movies were acquired in super-resolution counting mode (pixel size of 0.414 Å) at a nominal magnification of 105 000×, with defocus values ranging from −0.8 to −1.8 μm. Data were collected using a GIF energy filter with a 20 eV slit width. Automated data acquisition was performed using SerialEM at the Cleveland Center for Membrane and Structural Biology, Case Western Reserve University. Detailed acquisition parameters are provided in Supplementary Table S2.

### Cryo-EM data processing and model building

All cryo-EM data were processed using CryoSPARC v4.7.0 [62]. Patch-based motion correction and CTF estimation were performed for all micrographs, followed by initial quality filtering using the Junk Detector. Particle picking was carried out with the Blob Picker, and another round of particle picking was performed using 2D templates generated using the particles picked with Blob Picker. After performing 2D classification using the particles picked using Template Picker, Ab initio reconstructions were generated for both the nucleosome–scFv complex and junk classes, which were used as references for two rounds of heterogeneous refinement to further eliminate low-quality particles. The curated particle sets were refined using Non-Uniform Refinement, followed by Local CTF Refinement and reference-based motion correction to improve map quality. Subsequently, particles were subjected to 3D classification to resolve distinct conformational states. Classes corresponding to well-resolved nucleosome–protein complexes were selected and further refined using Local Refinement with a focused mask encompassing the bound proteins. The overall data-processing workflow is summarized in Supplementary Fig. S11 and Supplementary Table S3.

An initial structural model was generated using AlphaFold [63] and compared with a previously determined nucleosome–scFv complex (PDB: 7K61) [64] to assess overall consistency. The model was docked into the cryo-EM density using UCSF ChimeraX [65] and subjected to interactive refinement with the ISOLDE [66] plugin to improve local geometry and model–map fit. The structure was manually inspected and adjusted in COOT [67]. Iterative cycles of manual correction in COOT and real-space refinement in Phenix [68] were performed to optimize stereochemistry and agreement with the density. Final reconstruction and model statistics are provided in Supplementary Table S4.

## Results

### PARP2 specifically recognizes an SSB at SHL +5.3 in nucleosomal DNA

We first examined whether an N-terminally truncated PARP2 construct containing the WGR–CAT core (residues 90–583, Fig. 1A; hereafter PARP2) specifically recognizes a nucleosomal SSB positioned at a solvent-exposed site suitable for structural analysis. This construct was used for both the biochemical assays and cryo-EM experiments described below, as it partially retains DNA break-binding capacity through its WGR–CAT region despite lacking the N-terminal region, which also contributes to DNA binding, while showing reduced aggregation during cryo-EM sample preparation compared with full-length PARP2 [69, 70]. To this end, we reconstituted nucleosomes containing a defined SSB at SHL +5.3, where the DNA backbone faces outward from the histone surface (Fig. 1B). We selected SHL +5.3 because the nicked phosphate is positioned on the solvent-exposed face of the nucleosomal DNA, providing an accessible site for PARP2 interaction while preserving the nucleosome core for structural analysis. Although PARP2 interaction may differ at other SHLs depending on rotational exposure and proximity to histone surfaces, SHL +5.3 provided a tractable substrate to define how PARP2 recognizes an SSB embedded within nucleosomal DNA. The SSB was generated by incorporating a strand-specific Nt.BsmAI-R386D nicking site into the Widom 601 DNA and enzymatically nicking one strand before nucleosome reconstitution, producing a break with canonical 3′-OH and 5′-phosphate termini [59]. Complete nicking was confirmed by denaturing and native PAGE (Supplementary Fig. S1A), and the nicked DNA was purified by anion-exchange chromatography before nucleosome assembly [59].

**Figure 1.**
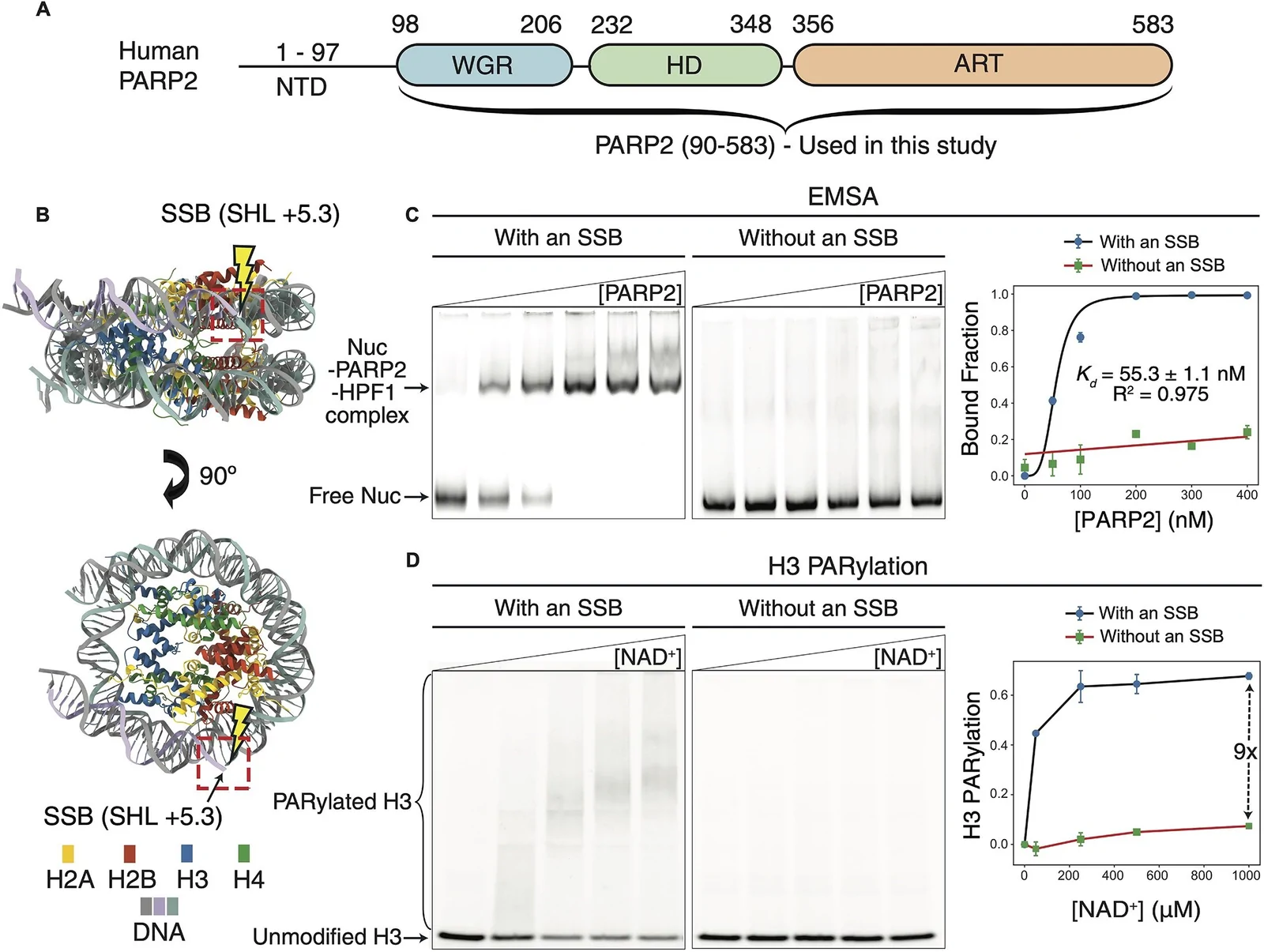
PARP2 specifically recognizes nucleosomal SSBs and promotes histone H3 PARylation.(**A**) Domain architecture of human PARP2 and the PARP2 construct used in this study, spanning residues 90–583 and containing the WGR, HD, and ART domains. (**B**) Cryo-EM structure of the nucleosome (PDB: 9YLR) containing an SSB at SHL +5.3, highlighting the position of the SSB (yellow lightning symbol in a red dashed box). Histone proteins are coloured as follows: H2A, H2B, H3, H4, and DNA. Two views related by a 90° rotation are shown. For clarity, the two scFv antibodies present in PDB: 9YLR have been omitted. (**C**) EMSA showing binding of PARP2 to a nucleosome containing an SSB at SHL +5.3. Increasing concentrations of PARP2 (0–400 nM) were incubated with 100 nM nucleosome in the presence of 5 µM HPF1 and analyzed by native PAGE. Nucleosomes lacking an SSB exhibit only non-specific binding. Quantification of the bound fraction is shown on the right, yielding an apparent dissociation constant (*K*_d_) of 55.3 ± 1.1 nM. Error bars represent the SD from duplicate experiments. (**D**) Histone H3 PARylation assay using nucleosomes containing an SSB at SHL +5.3. Increasing concentrations of NAD^+^ (0–1 mM) were added to reactions containing 1 µM PARP2 and 0.2 µM nucleosome substrate in the presence of 3 µM HPF1. Histone H3 PARylation is strongly stimulated in the presence of the SSB, whereas intact nucleosomes show markedly reduced modification. Quantification of H3 PARylation as a function of NAD^+^ concentration is shown on the right. Error bars represent the SD from duplicate experiments.

EMSA performed in the presence of HPF1 showed that PARP2 binds efficiently to nucleosomes containing the SSB, yielding an apparent dissociation constant (*K*_d_) of ∼55.3 nM (Fig. 1C). In contrast, intact nucleosomes lacking an SSB showed only weak binding under the same conditions (Fig. 1C). When assayed under identical conditions, free DNA containing the same SSB was bound with substantially lower affinity (*K*_d_ = 653 nM), indicating that PARP2 preferentially recognizes the SSB in the nucleosomal context (Supplementary Fig. S1B and C). To assess the contribution of HPF1, we performed an additional EMSA under matched conditions in the absence of HPF1. Comparison of the two assays showed comparable apparent binding affinities, suggesting that HPF1 does not substantially alter PARP2 association with the nucleosomal SSB under these conditions (Supplementary Fig. S1D, bottom). However, the presence of HPF1 produced a supershift of the PARP2-bound nucleosome relative to PARP2 alone, consistent with formation of a nucleosome–PARP2–HPF1 complex (Supplementary Fig. S1D, top).

We next examined whether PARP2 binding at this site supports catalytic activity. The nucleosome containing an SSB at SHL +5.3 showed enhanced histone H3 PARylation relative to intact nucleosomes, consistent with the preferential binding detected by EMSA (Fig. 1D). In contrast, H3 PARylation was not detected in reactions lacking HPF1, confirming that histone modification under these conditions is HPF1-dependent as reported previously (Supplementary Fig. S2A) [71]. Notably, intact nucleosomes also showed small amount of H3 PARylation despite showing only weak nonspecific binding by EMSA (Fig. 1D). This may reflect the enzymatic nature of the PARylation assay, which can amplify transient or low-affinity PARP2–nucleosome interactions into a detectable catalytic signal. In addition to H3 modification, PARylation of PARP2 and HPF1 was also enhanced with SSB-bearing nucleosomes compared with intact nucleosomes (Supplementary Fig. S2B, left). We further compared free duplex DNA substrates with or without the same SSB. SSB-containing free DNA similarly enhanced PARylation of PARP2 and HPF1, although basal PARylation was detectable at high NAD^+^ concentrations even without an SSB (Supplementary Fig. S2B, right). Together, these results indicate that SSBs enhance PARP2–HPF1 catalytic activity in both nucleosomal and free DNA contexts, while the nucleosomal SSB at SHL +5.3 promotes efficient PARylation of H3, PARP2 and HPF1. Based on these findings, we selected this substrate for cryo-EM structural analysis.

### Cryo-EM structures reveal PARP2–HPF1 binding to a nucleosomal SSB at SHL +5.3

To elucidate the structural basis of PARP2 activity in nucleosome, we sought to determine cryo-EM structures of PARP2 and HPF1 bound to a nucleosome containing an SSB at SHL +5.3. Despite extensive optimization of sample and vitrification conditions, we were initially unable to detect nucleosome particles with PARP2 and HPF1, suggesting disruption of the complex at the air–water interface. To overcome this, we employed the scFv derived from the PL2-6 antibody, which is known to bind the acidic patch and stabilize nucleosomes for cryo-EM [61, 72]. Another key factor enabling structural determination was the use of the truncated PARP2 mutant construct (residues 90–583) lacking the disordered N-terminal domain, as the full-length PARP2 formed aggregation on the grid (Fig. 1A). Using this approach, we successfully determined the structure of the nucleosome with an SSB at SHL +5.3 in complex with PARP2 and HPF1 at 2.8 Å resolution (Fig. 2A and Supplementary Fig. S3A–C). The overall resolution mainly reflects the well-ordered nucleosome core and scFv, whereas the PARP2–HPF1 region showed lower local resolution, with the WGR, HD, ART, and HPF1 resolved to ∼3–7 Å (Supplementary Fig. S3A–C). Notably, the scFvs do not interact with nucleosomal DNA or with the PARP2–HPF1 complex, and binding affinities measured in the absence and presence of scFv are comparable (Fig. 2A and Supplementary Fig. S1E).

**Figure 2.**
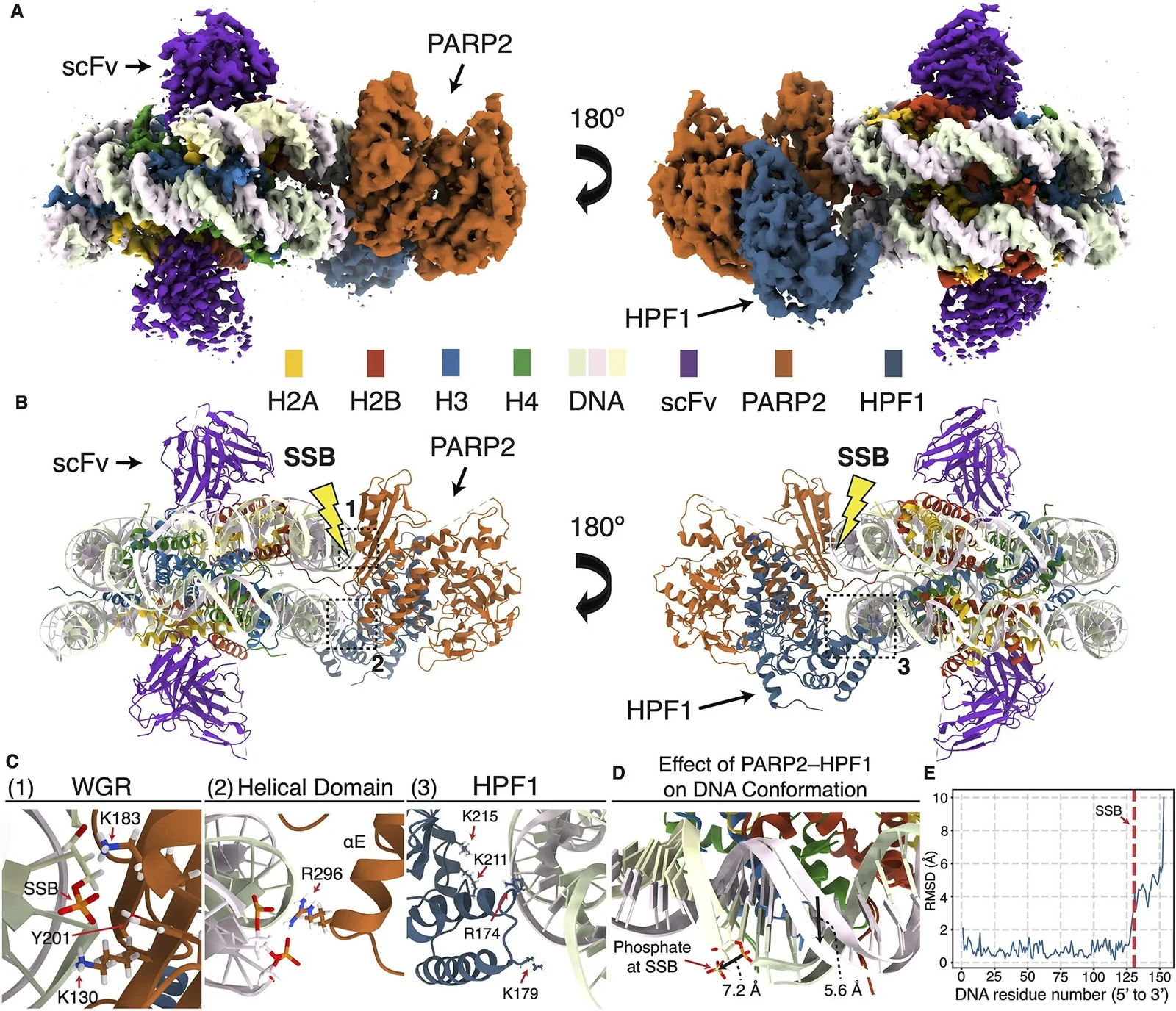
Cryo-EM structure of PARP2–HPF1 bound to a nucleosomal SSB reveals multivalent DNA engagement and PARP2-induced DNA deformation. (**A**) Cryo-EM density map of the nucleosome containing an SSB at SHL +5.3 bound by PARP2–HPF1, shown in two orientations related by a 180° rotation. Components are coloured as indicated. The scFv binds the acidic patch and does not contact the nucleosomal DNA or PARP2–HPF1. (**B**) Atomic model of the PARP2–HPF1–nucleosome-scFv complex (PDB: 9ZTN) shown in two orientations. The lightning-bolt icon marks the SSB at SHL +5.3. Three major DNA-interaction sites are indicated: (1) the PARP2 WGR domain engages the SSB on the upper DNA gyre; (2) Arg296 in the HD contacts the lower DNA gyre; and (3) HPF1 contacts the lower DNA gyre. (**C**) DNA contact sites: (1) the WGR domain binds the SSB on the upper DNA gyre; (2) Arg296 on helix αE contacts the lower gyre; (3) HPF1 binds the lower gyre via basic residues. (**D**) Overlay of DNA trajectories from the unbound nucleosome and the PARP2–HPF1-bound nucleosome at the SSB site, highlighting PARP2–HPF1-induced DNA deformation. Arrows indicate the displacement between the two DNA conformations near the SSB. (**E**) Per-nucleotide DNA RMSD relative to the nucleosome alone, highlighting deviations at and downstream of the SSB.

The structure reveals how PARP2 and HPF1 engage an SSB within nucleosomal DNA through multiple contact points on a single nucleosome (Fig. 2A–C). The WGR domain recognizes the break on the upper DNA gyre through conserved contacts, while Arg296 on helix αE of the HD and basic residues in HPF1 contact the lower gyre, together spanning both DNA gyres and the inter-gyre space (Fig. 2B and C and Supplementary Fig. S4A–E). Consistent with this interaction, sequence alignment of HPF1 orthologs showed that the positively charged character of this DNA-facing surface, including residues corresponding to human R174, K179, K211, and K215, is broadly conserved across vertebrates (Supplementary Fig. S5). This multivalent binding configuration distorts the DNA backbone at the break site, displacing the phosphate of the broken strand by 7.2 Å and the complementary strand by 5.6 Å relative to the nucleosome structure containing an SSB at SHL +5.3 determined in the absence of PARP2–HPF1 (Fig. 2D and Supplementary Movie S1). These distortions propagate into the linker DNA, indicating that the structural perturbation extends beyond the break site, changing the nucleosomal DNA trajectory, and indicating that PARP2-HPF1 binding potentially alters local nucleosomal DNA structure (Fig. 2E). These coordinated interactions explain the higher affinity of PARP2–HPF1 for nucleosomal SSBs compared with free DNA and highlight a binding mode uniquely adapted to the geometric constraints of nucleosome.

### PARP2 samples multiple DNA-bound states at an SHL +5.3 nucleosomal SSB

Further analysis of the cryo-EM dataset suggested that PARP2–HPF1 does not bind nucleosomal SSBs in a single static conformation. Instead, 3D classification resolved ten particle classes with distinct PARP2 and DNA conformations (Fig. 3A and B). These classes can be grouped into three binding modes based on the position of a helix within the WGR domain (residues 169–183), providing snapshots of alternative PARP2–DNA arrangements (Fig. 3A). In binding mode 1, represented by Classes 1–3, the WGR domain adopts a conserved conformation (Fig. 3A, left). Class 1 contains density for the full PARP2–HPF1 assembly, whereas Class 2 retains the WGR domain and parts of the HD, and Class 3 shows density only for the WGR domain, showing progressively increased dynamics. In Class 2, density for the ART domain, HPF1, and helices αA, αB, and part of αF (residues 339–347) is lost, suggesting destabilization of these regions (Fig. 3A, inset). This behaviour agrees with previous HDX-MS data showing increased exchange in helices αF and αB upon DNA binding [53, 58]. Although high-resolution features of the destabilized regions cannot be resolved, low-resolution density suggests disruption of interactions between the active site loop (ASL) and helices αD and αF, showing opening of the ART domain that may allow NAD^+^ binding (Supplementary Fig. S4G and H).

**Figure 3.**
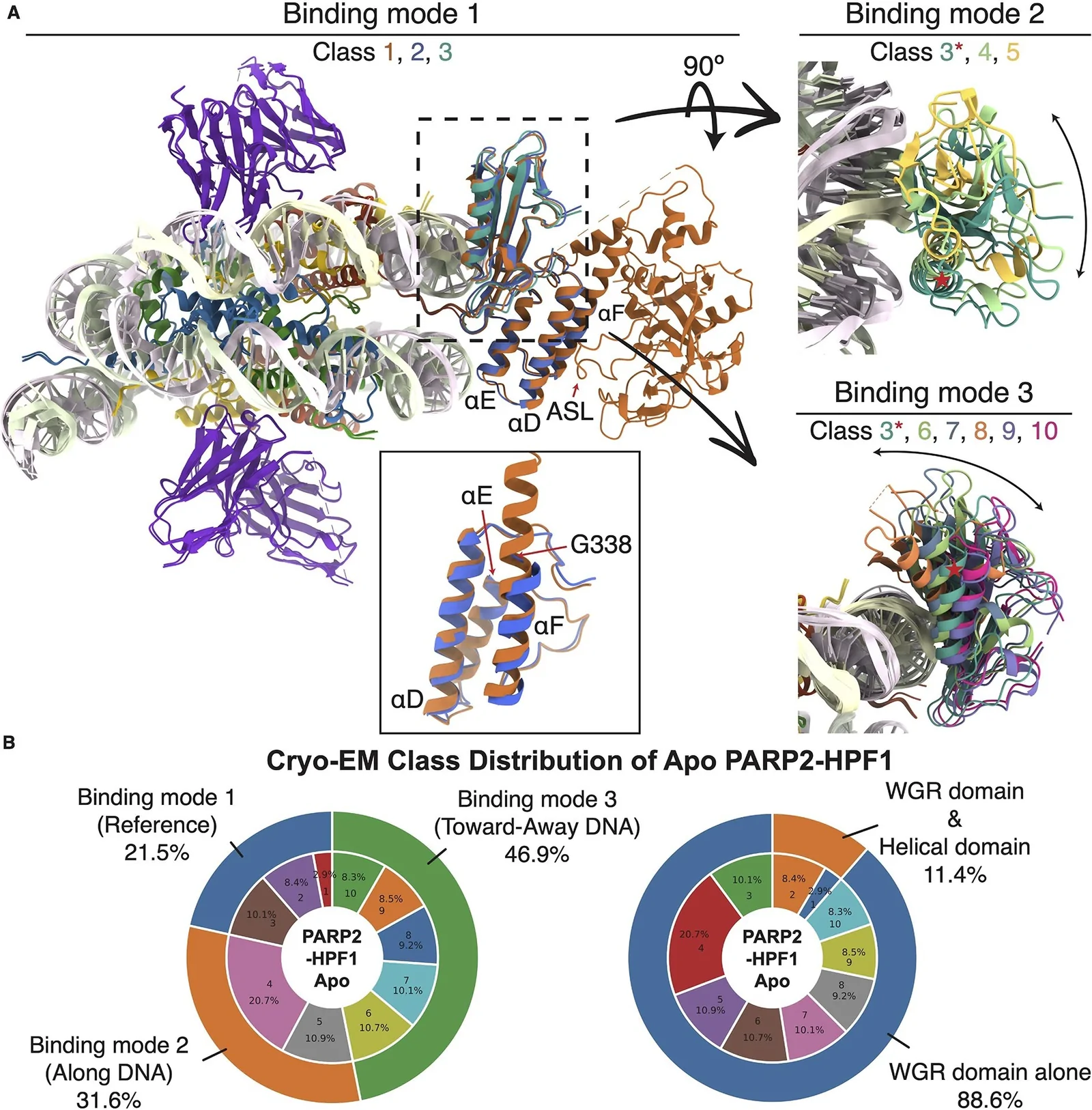
Cryo-EM classification reveals multiple PARP2–HPF1 binding modes at a nucleosomal SSB. (**A**) Overview of the complete apo PARP2–HPF1–nucleosome complex in the binding mode 1 orientation and comparison of three binding modes defined by WGR-domain position. Inset showing enlarged superposition of the HD αD–αF helices from Classes 1 and 2, with the position of G338 indicated. Binding mode 1 includes Classes 1–3 and shows a conserved WGR–DNA arrangement with progressive loss of PARP2–HPF1 density. Binding mode 2 includes Classes 4 and 5 and shows lateral movement of the WGR domain along DNA, whereas binding mode 3 includes Classes 6–10 and shows movement of the WGR helix toward or away from nucleosomal DNA. Class 3, marked with a red star, is included as a reference in binding modes 2 and 3. (**B**) Particle distribution of apo PARP2–HPF1 cryo-EM classes. Left, classes are grouped into three binding modes, with the inner ring showing individual class populations and the outer ring showing grouped binding modes. Right, particles classified by the presence of WGR-domain density alone or both WGR and HD density.

This heterogeneity is also reflected in the particle distribution: only ∼11.4% of particles retain density for both the WGR and HD (Classes 1 and 2), and only ∼2.9% adopt the fully assembled WGR–HD–ART architecture in complex with HPF1 (Class 1) (Fig. 3B, right). In the remaining classes (Classes 3–10), density is restricted to the WGR domain, indicating destabilization of its interactions with the HD. In binding mode 2, represented by Classes 4 and 5, the WGR domain shifts laterally along the DNA, accompanied by concerted movement of the DNA itself (Fig. 3A, top right, and Supplementary Movie S2). In Class 5, nucleotides at the SSB exhibit RMSDs of up to 6.7 Å, with the complementary strand displaced by up to 4.7 Å relative to binding mode 1 (Supplementary Fig. S4I). Across the classes within binding mode 2, these structural changes reflect shifts in DNA register accompanied by downstream distortion of the duplex, occurring in different directions and magnitudes. Class 4 adopts an intermediate conformation between Classes 3 and 5, possibly an on-pathway transition toward the Class 5 conformation.

In binding mode 3, represented by Classes 6–10, a helix within the WGR domain moves toward or away from the nucleosomal DNA (Fig. 3A, bottom right, and Supplementary Movie S3). Class 8 displays the largest rearrangements, with DNA backbone shifts of up to 5.5 Å relative to binding mode 1 (Supplementary Fig. S4J), whereas Classes 6 and 7 show more modest changes consistent with intermediate states. In Classes 9 and 10, the same WGR helix shifts away from the DNA, accompanied by smaller structural deviations. As in binding mode 1, these WGR rearrangements are coupled to corresponding distortions at the SSB site (Supplementary Fig. S4J). Together, these binding modes suggest that PARP2 can sample multiple DNA-bound states on nucleosomal SSBs, with each state associated with distinct local DNA distortions. These observations support a model in which PARP2 uses structural plasticity to engage SSBs within diverse chromatin geometries.

### Nucleosomal geometry reorganizes PARP2–HPF1 architecture

Comparison with previous PARP2 structures revealed both conserved and context-dependent features of DNA-break recognition [18, 56, 57]. The WGR domain in our nucleosomal SSB-bound structure closely resembles the WGR conformations observed in previous PARP2 structures determined with free DNA, linker DNA, or nucleosome-proximal DNA breaks (PDB IDs: 7AEO, 6X0N, and 6USJ), indicating that the core WGR-mediated mode of DNA-break recognition is largely preserved across different DNA contexts (Supplementary Fig. S6A). In contrast, the HD and ART domains show greater positional variability. When aligned to the WGR domain of the PARP2 structure bound to free DNA (PDB ID: 7AEO), the ART domain in our structure is displaced by ∼30–42 Å, whereas helices αA and αB of the HD shift by ∼25–34 Å, depending on the reference points used for measurement (Supplementary Fig. S6B–D). Notably, the C-terminal segment of helix αF (residues 339–354) is not resolved in the free-DNA structure, consistent with the idea that this region is intrinsically flexible and may allow large-scale repositioning of the HD and ART domains between free-DNA- and nucleosome-bound states.

When compared with the PARP2–HPF1 structure (PDB: 6X0N) solved on a DSB in linker DNA [18], our nucleosomal SSB structure reveals features of DNA interaction and domain organization that arise from engaging an SSB within the nucleosomal DNA (Supplementary Fig. S6E). When the two structures are aligned using the WGR domain, we observed two key differences. A difference occurs at the end of helix αE in the HD: in our structure, Arg296 contacts the nucleosomal lower gyre, resulting in a ∼1.0 Å shift of the helix away from the DNA compared with the previous structure (PDB ID: 6X0N), where PARP2–HPF1 binds linker DNA and does not engage a nucleosomal gyre (Supplementary Fig. S6E, left). More importantly, we observe shifts in the ART domain by 3.0–4.3 Å and HPF1 by 2.9–6.8 Å depending on the reference points (Supplementary Fig. S6E, right). These shifts in the ART domain and HPF1 likely originate from a shift in the C-terminal part of the helix αF in the HD spanning residues 339–347 which is displaced by 2.0 Å (Supplementary Fig. S6E, left). This again reflects the intrinsic dynamics of this segment of helix αF, which enable the ART domain and HPF1 to sample multiple conformations.

### HPF1 Reshapes the conformational landscape of PARP2 on nucleosomes

To determine how HPF1 reshapes the conformational landscape of PARP2 on SSB-containing nucleosomes, we next analyzed a corresponding cryo-EM dataset collected in the absence of HPF1 and compared it with the PARP2–HPF1 dataset described above. In the PARP2-only dataset, 3D classification yielded 14 particle classes spanning three binding modes similar to those observed in the PARP2–HPF1 complex (Fig. 4A and D, compare Fig. 4D, top with Fig. 3B, left). The WGR domain engaged nucleosomal DNA through comparable contacts in both datasets. However, the distribution of resolved domains differed significantly. In the absence of HPF1, 43.6% of particles retained density for both the WGR and HD, whereas 56.4% resolved only the WGR domain (Fig. 4D, bottom). In contrast, the PARP2–HPF1 dataset was dominated by classes in which only the WGR domain was resolved (88.6%), with a smaller fraction of particles retaining density for both the WGR and HD (11.4%) (compare Fig. 3B, right with Fig. 4D, bottom). Structural overlays further revealed pronounced conformational variability across helices αD, αE, and αF of the HD in the PARP2-only structures, in contrast to the PARP2–HPF1 structure, in which only a single HD conformation was detected (Fig. 4B). Among these variable regions, helix αF showed the largest displacement, shifting by up to ∼12 Å relative to the PARP2–HPF1 reference structure (Fig. 4C). Notably, none of the PARP2-only classes showed resolvable density for the ART domain, indicating that the catalytic domain remains highly dynamic in the absence of HPF1. Together, these observations suggest that HPF1 reshapes the conformational ensemble of PARP2 on nucleosomes.

**Figure 4.**
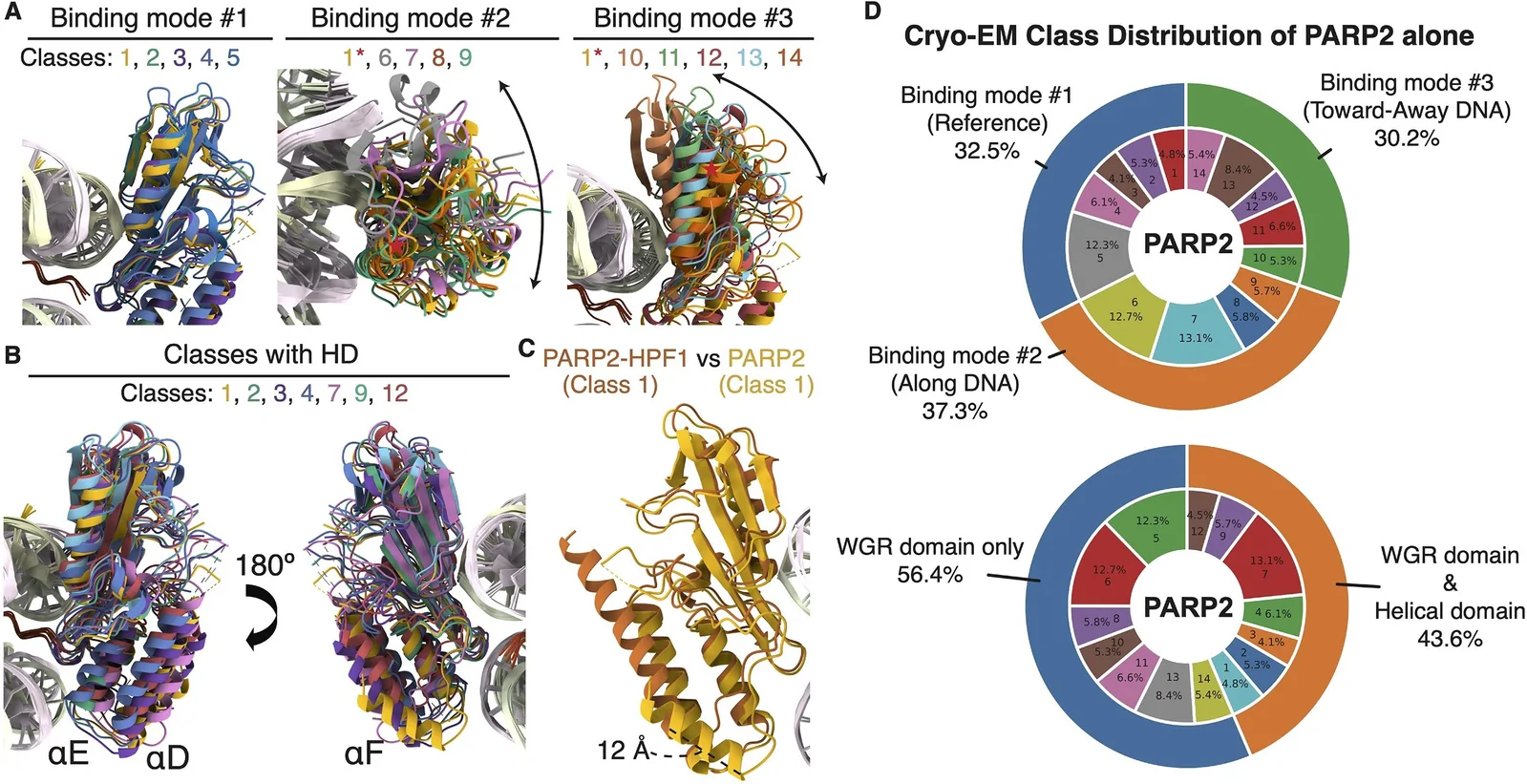
Cryo-EM analysis of PARP2 bound to nucleosomes in the absence of HPF1 reveals extensive conformational heterogeneity. (**A**) Structural overlays of PARP2 from representative cryo-EM classes grouped into three binding modes observed in the PARP2-only dataset. The WGR domain engages nucleosomal DNA through similar contacts across classes, as observed in the PARP2–HPF1 dataset, while the relative orientation of downstream domains varies. (**B**) Structural overlays highlighting conformational variability of the PARP2 HD. Major differences are observed across helices αD, αE, and αF of HD. Two orientations related by a 180° rotation are shown. (**C**) Structural comparison of Class 1 PARP2–HPF1 and PARP2 alone, illustrating displacement of helix αF of up to ∼12 Å in the absence of HPF1. (**D**) Cryo-EM class distribution for the PARP2-only dataset. The upper chart shows particle populations grouped by binding mode. The lower chart summarizes the proportion of classes resolving only the WGR domain versus those resolving both the WGR and HD, highlighting the structural heterogeneity of PARP2 in the absence of HPF1.

### The WGR domain alone does not confer PARP2 plasticity

To determine whether the WGR domain alone accounts for the conformational heterogeneity observed in PARP2, we solved the cryo-EM structure of the isolated WGR domain bound to a nucleosome containing an SSB at SHL +5.3 (Supplementary Fig. S7A and C). In contrast to PARP2 or PARP2–HPF1, which sample a broad ensemble of DNA-bound states, the isolated WGR domain adopted only two closely related conformations, indicating a relatively rigid mode of DNA binding (Supplementary Fig. S7B). These results suggest that the conformational heterogeneity observed for PARP2 is linked to mobility of the HD and ART domains rather than to large intrinsic rearrangements of the isolated WGR domain.

### PARPis stabilize DNA binding but increase ART–HPF1 dynamics

To assess how PARPis alter PARP2 structure on nucleosomes, we determined cryo-EM structures of the PARP2–HPF1–nucleosome complex in the presence of olaparib (Fig. 5A). Although the apo PARP2–HPF1 dataset, prepared without PARPi, is conformationally heterogeneous, a minor subset of particles (2.9%) retained ordered density for the ART domain and HPF1 in a defined architecture. In contrast, the olaparib-bound dataset did not yield classes with comparably ordered ART–HPF1 density suitable for model building. Because the ART–HPF1 module was highly dynamic in this dataset, olaparib density could not be assigned unambiguously in the cryo-EM maps. We therefore confirmed inhibitor engagement using PARylation activity assays with the same PARP2, HPF1 and nucleosome constructs used for cryo-EM, in which increasing concentrations of olaparib reduced PARylation of histone H3, PARP2, and HPF1 in a concentration-dependent manner (Supplementary Fig. S8). In the cryo-EM reconstructions, weak residual density attributable to the ART–HPF1 module was distributed across multiple positions near both DNA gyres, whereas the WGR and HD remained resolved (Fig. 5A–C). Thus, although the ART–HPF1 module is already dynamic in the apo state, olaparib binding appears to further disfavour a stable, well-ordered ART–HPF1 configuration, enabling broader motions that sample both DNA gyres while retaining ordered density for the WGR–HD region. This correlates with the loss of density in the C-terminal region of helix αF in the presence of olaparib, a segment that governs the orientation of the ART–HPF1 module (Fig. 5B, middle).

**Figure 5.**
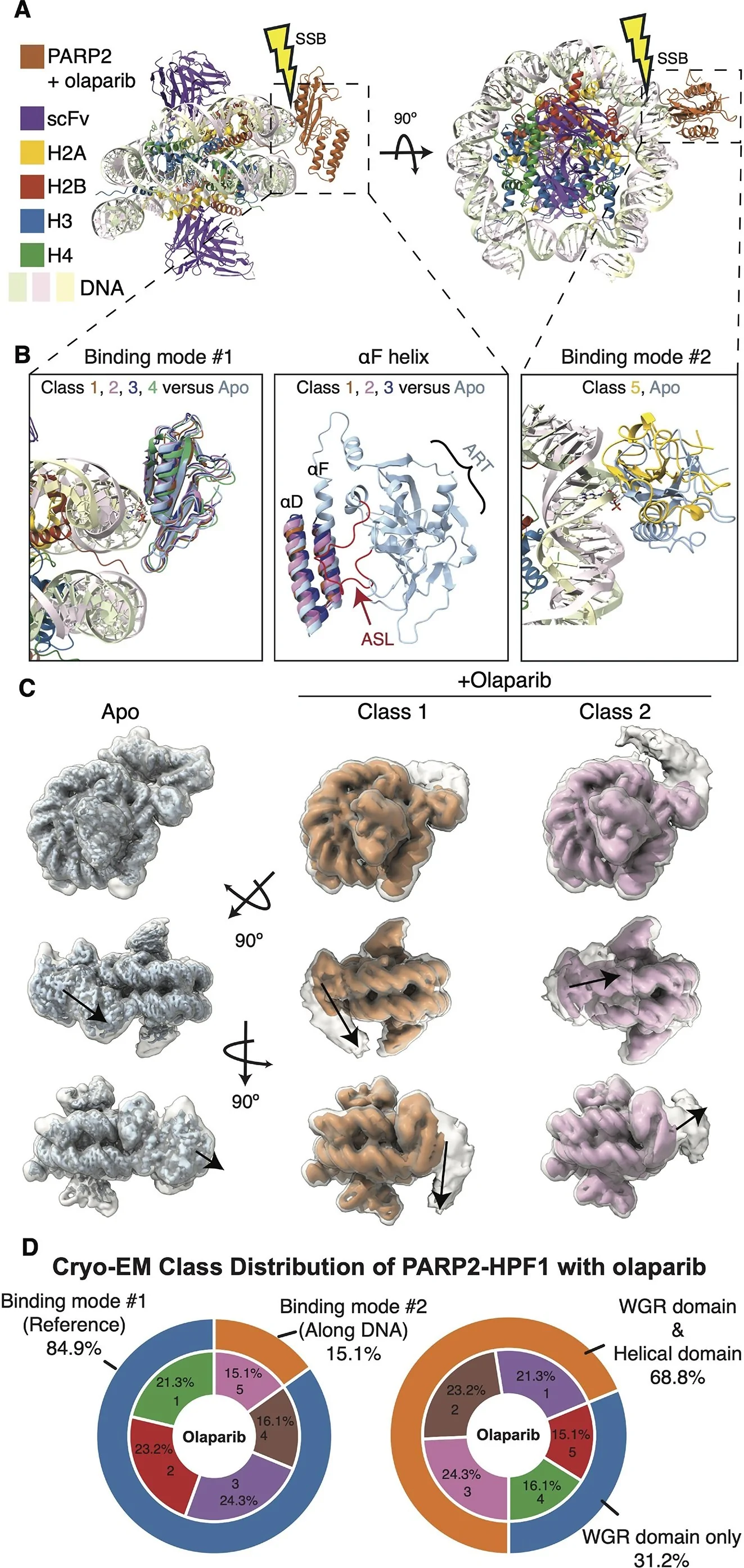
Olaparib enhances PARP2 conformational dynamics of ART-HPF1 domains but stabilizes WGR-HD domains. (**A**) Cryo-EM structure of PARP2 bound to a nucleosome containing an SSB at SHL +5.3 in the presence of olaparib, shown in two orthogonal views. PARP2, scFv, histones, and DNA are indicated; the SSB is marked by a lightning bolt. (**B**) Left, binding mode 1 shows a conserved PARP2 orientation relative to DNA. The WGR domain is shown only for clarity. Middle, structural overlays reveal olaparib-induced loss of ordered density in the C-terminal helix αF and disruption of contacts with the ASL. Right, the WGR domain in binding mode 2 (Class 5). The Class 1 of PARP2-HPF1 in apo form is shown in light blue. (**C**) Density maps of apo and olaparib-bound PARP2–HPF1 classes (Classes 1 and 2) shown in multiple orientations, illustrating increased mobility of the ART domain and HPF1 upon inhibitor binding. (**D**) Distribution of cryo-EM particle classes for the olaparib-bound PARP2–HPF1–nucleosome dataset, categorized by binding mode and domain visibility, showing a redistribution of particle populations relative to the apo complex.

In parallel, conformational variability within the WGR domain was markedly reduced relative to the apo dataset. The majority of particles adopted binding mode 1 (84.9%), with only a minor class occupying binding mode 2 (15.1%) (Fig. 5D, left). Notably, the proportion of classes in which both the WGR and HD were resolved increased significantly (Fig. 5D, right). Olaparib binding thus restricts WGR conformational heterogeneity (compare Fig. 5D, left, with Fig. 3B, left) while stabilizing states in which the WGR and HD domains are jointly resolved (compare Fig. 5D, right, with Fig. 3B, right), suggesting increased coupling between these domains upon inhibitor binding. Together, these findings indicate that olaparib-bound PARP2 favours states with more stable WGR–DNA and WGR–HD interfaces, while the ART–HPF1 catalytic module exhibits greater positional variability relative to the WGR domain and the bound nucleosome.

### PARPis promote nucleosomal DNA unwrapping

In addition to its effects on PARP2, olaparib also remodels nucleosome structure. Three classes showed partially unwrapped states in which DNA peels from the histone octamer on the PARP2–HPF1-bound side, while the opposite arm remains fully wrapped (Fig. 6A). These states retain 136, 132, or 127 bp of wrapped DNA, while the apo dataset contains only ∼9% of particles with 136 bp of wrapped DNA (Fig. 6A and B, and Supplementary Fig. S9A). Together, these observations indicate that olaparib-bound PARP2 destabilizes local histone–DNA contacts and promotes asymmetric DNA unwrapping at the binding interface. Cryo-EM structures with talazoparib and EB-47 showed overall effects similar to those observed with olaparib. Despite differences in class populations, all inhibitors shifted the nucleosome ensemble toward partially unwrapped DNA conformations, reflecting a shared modulation of nucleosomal DNA geometry (Supplementary Fig. S9B and C). Importantly, free nucleosome particles present in the same inhibitor-bound datasets (olaparib, talazoparib and EB-47) showed only ∼11%–13% partial unwrapping, comparable to previously reported levels of spontaneous nucleosome unwrapping and to the level observed in the apo dataset in this study (Supplementary Table S3) [73]. Thus, inhibitor-associated nucleosome unwrapping is coupled to PARP2–HPF1 binding, rather than arising from a direct, global effect of PARPis on nucleosome structure.

**Figure 6.**
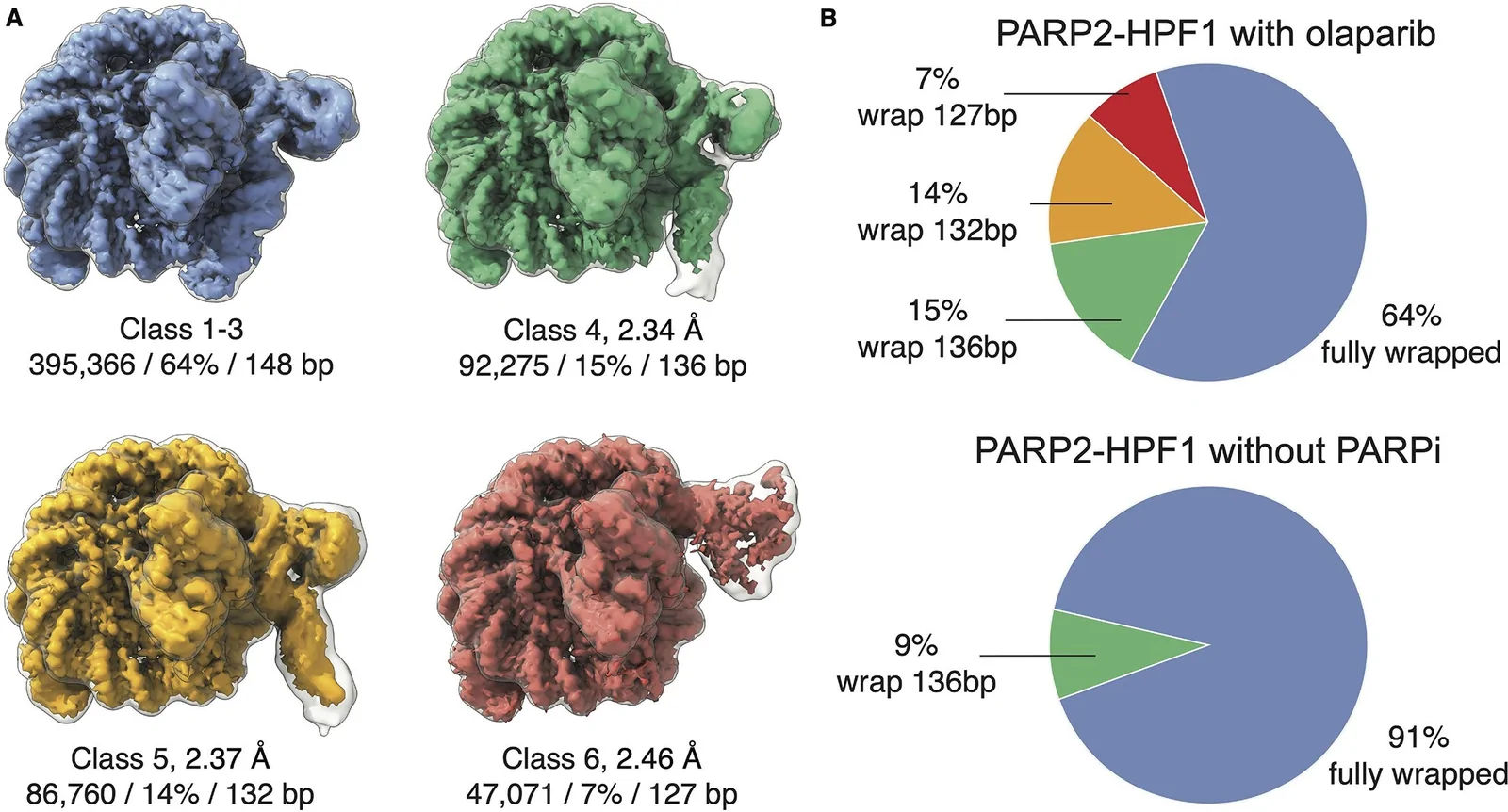
Partial unwrapping of nucleosomal DNA in the presence of olaparib. (**A**) Representative cryo-EM maps of PARP2–HPF1–nucleosome complexes in the presence of olaparib showing individual particle classes. Particle numbers, relative populations, map resolutions, and the number of base pairs of wrapped DNA are indicated for each class. Classes corresponding to partially unwrapped nucleosomal DNA (136 bp, 132 bp, and 127 bp wrapped) are observed in addition to the fully wrapped state (148 bp). (**B**) Distribution of nucleosomal DNA wrapping states across cryo-EM classes. In the presence of olaparib, multiple partially unwrapped nucleosome states are observed. In contrast, the apo PARP2–HPF1 dataset (without PARPi) is dominated by the fully wrapped nucleosome state, with only a minor population of partially unwrapped DNA.

Talazoparib and EB-47, like olaparib, reduced WGR-domain conformational variability relative to the apo complex, while the ART domain and HPF1 remained highly dynamic across all inhibitor datasets (Supplementary Figs S9D and S10C). In addition, similar to olaparib, the majority of particles in the talazoparib- and EB-47-bound datasets show density for both the WGR and HD domains (Supplementary Fig. S10A–C). Although the C-terminal region of helix αF was largely disordered in olaparib- and talazoparib-bound complexes, it was retained in a subset of EB-47-bound classes, indicating that distinct inhibitors can differentially modulate local dynamics within the HD (Supplementary Fig. S10B, right). Together, these results indicate that the three PARPis tested in this study induce a common structural response characterized by reduced conformational heterogeneity of the WGR domain and stabilization of WGR–HD interactions. Because olaparib, talazoparib, and EB-47 have each been linked to PARP2 retention, stabilization of these interfaces may provide a structural explanation for this shared inhibitor-dependent retention phenotype [74, 75]. At the same time, they increase the flexibility of the catalytic module and promote asymmetric nucleosomal DNA unwrapping, collectively reshaping PARP2–nucleosome interactions.

## Discussion

Although PARP2 plays a critical role in responding to single-strand DNA breaks [76–80], the structural mechanisms by which it recognizes lesions within chromatin have remained unclear. Here, cryo-EM structures of PARP2 bound to nucleosomes containing an SSB within the nucleosome core show how chromatin architecture shapes PARP2 interaction with damaged DNA. Cryo-EM structures reveal that PARP2 and HPF1 recognize nucleosomal SSBs through three coordinated interaction points: the WGR domain at the SSB phosphate, Arg296 in helix αE of HD, and HPF1 at the lower DNA gyre. This multivalent binding configuration allows PARP2 to engage lesions within the geometric constraints of the nucleosome and explains its higher affinity for nucleosomal SSBs relative to free DNA.

3D classification revealed multiple DNA-bound states that differ in WGR orientation, as well as in the structural stability and conformation of downstream PARP2 domains. These classes are associated with distinct DNA backbone distortions near the break, suggesting that PARP2 binding can be accommodated by several nucleosomal DNA conformations. Such structural plasticity likely enables PARP2 to accommodate SSBs embedded within diverse chromatin geometries and DNA topologies. The structural perturbation generated by PARP2 extends beyond the break site along the downstream DNA and into the linker region, where it may influence local chromatin organization.

It is worth noting that the specific position of the SSB within the nucleosome likely dictates PARP2 interaction. Because rotational exposure of the damaged strand, proximity of the 5′-phosphate to the histone surface, and spacing between the break and the opposing DNA gyre vary across SHLs, SSBs at alternative nucleosomal sites may favour distinct WGR orientations, DNA distortions, or HD conformations. Thus, the SHL +5.3 structure represents just one accessible nucleosomal SSB geometry. Future studies comparing additional lesion positions will be essential to determine how location tunes PARP2 binding, activation, and chromatin retention.

Our data further reveal an important structural role for HPF1 in organizing the catalytic region of PARP2 on nucleosomes. In the presence of HPF1, the conformational ensemble of PARP2 shifts toward states in which the HD adopts more defined conformations likely compatible with histone PARylation, consistent with the established requirement of HPF1 for histone ADP-ribosylation [32]. In contrast, PARP2 alone exhibits greater conformational variability, with pronounced heterogeneity across helices αD, αE, and αF of HD and no resolvable density for the ART domain. Together, these observations suggest that HPF1 functions not only as a catalytic cofactor but also as a structural organizer that promotes configurations of PARP2 compatible with histone modification on chromatin.

The cryo-EM structures also provide insight into how PARPis influence PARP2–chromatin interactions through reverse allostery–allosteric communication from the catalytic ligand-binding site back to distal DNA-binding interfaces [81, 82]. In the apo PARP2–HPF1 complex, PARP2 samples both closed and open catalytic-pocket conformations, with the open state associated with destabilization of the C-terminal region of helix αF within the HD, likely facilitating ART domain opening and access of NAD^+^ or PARPis to the catalytic pocket. Upon PARPi binding, the ART–HPF1 catalytic module becomes highly dynamic while the WGR and HD DNA-binding interfaces remain stabilized on nucleosomal DNA. This redistribution—characterized by stabilization of the DNA-binding scaffold (WGR and parts of HD) with increased ART-HPF1 dynamics—is consistent with reverse allosteric coupling, whereby binding at the catalytic site is communicated outward to remodel distal DNA-binding interfaces and promote PARP retention at breaks.

Our structures suggest that inhibitor binding does not globally rigidify the PARP2–HPF1 complex. Instead, PARPis appear to selectively stabilize the WGR–DNA and WGR–HD interfaces while the ART–HPF1 module remains conformationally dynamic. Although stabilizing the WGR–HD region and its associated DNA contacts impose an entropic cost, this penalty could be partially offset by the increased flexibility of the ART–HPF1 module, rendering this state energetically favourable for retention on damaged chromatin. Thus, the dynamics of the ART–HPF1 module is unlikely to drive PARP retention through direct interactions; rather, its dynamics may facilitate PARP2 to maintain stable engagement with the nucleosomal break without global stabilization of the entire PARP2–HPF1 assembly. This interpretation is also consistent with the limited contribution of HPF1 to apparent PARP2 binding under our EMSA conditions, suggesting that inhibitor-dependent retention is primarily encoded in the PARP2 WGR–DNA and WGR–HD interfaces rather than in HPF1-mediated DNA contacts. Together, these observations support a model in which PARPis promote a retention-compatible state by stabilizing the DNA-bound PARP2 scaffold while redistributing conformational dynamics to the ART–HPF1 catalytic module.

A recent biochemical study showed that the three PARPis used here—olaparib, talazoparib, and EB-47—allosterically decrease the dissociation rate of PARP2 from DNA in addition to inhibiting catalysis [74]. Consistently, olaparib and talazoparib have been shown to promote PARP2 retention on DNA in cells [75]. For PARP2, all three inhibitors used in this study are therefore classified as Type I pro-retention agents, whereas Type II inhibitors have little or no effect on retention and Type III inhibitors promote dissociation. Importantly, however, these classifications are PARP-specific: among the inhibitors examined here, only EB-47 strongly promotes PARP1 retention, whereas olaparib and talazoparib have little or no pro-retention effect on PARP1 [74, 82–84]. Consequently, these three compounds used in this study should not be taken to represent all possible PARPi allosteric mechanisms; other inhibitors with distinct chemotypes or trapping classes may impose different effects on the HD, αF region, ART–HPF1 mobility, and WGR–HD coupling. Together, these insights indicate that the structural differences we observe between inhibitor-free and inhibitor-bound PARP2–HPF1–nucleosome complexes likely reflect inhibitor-dependent remodeling of the PARP2 allosteric network, providing a clear mechanistic basis for the enhanced retention of PARP2 on damaged chromatin.

Beyond PARP2 retention at the break, inhibitor binding also promotes partial unwrapping of nucleosomal DNA, revealing a structural mechanism by which PARPis can facilitate remodeling of chromatin structure at sites of DNA damage. Nucleosomal DNA normally undergoes spontaneous “breathing,” in which outer DNA segments transiently unwrap and rewrap from the histone core due to thermal fluctuations, reflecting the intrinsic flexibility of histone–DNA contacts [85–88]. Increased DNA unwrapping may result from PARPis stabilizing PARP2 on DNA and prolonging its residence at SSBs [74, 75]. Extended retention of PARP2 at the lesion is expected to restrict local nucleosome flexibility, thereby constraining normal histone–DNA dynamics. As a consequence, nucleosome breathing is biased toward more extensive DNA unwrapping, which may serve as a mechanism to relieve accumulated torsional or bending strain within the nucleosomal DNA. In this model, PARPis may indirectly alter local nucleosomal DNA structure by PARP2 retention at damage sites and shifting the dynamic equilibrium toward partially unwrapped nucleosome states. If a similar shift occurs in cells, it could transiently expose nucleosomal entry–exit DNA, alter linker-DNA trajectory and nucleosome–nucleosome packing, and increase accessibility to repair factors near the break.

Together, these structures establish a structural framework for understanding how PARP2 recognizes DNA breaks within chromatin and how PARPis reshape PARP2–chromatin interactions. By revealing the conformational landscape of PARP2 on nucleosomal substrates, our findings provide insight into the structural basis of PARP2 activation, HPF1-mediated regulation, and PARPi action in the chromatin environment.

## Supplementary Material

gkag939_Supplemental_Files

## Data Availability

Cryo-EM maps generated in this study have been deposited in the Electron Microscopy Data Bank (EMDB), and the corresponding atomic models have been deposited in the Protein Data Bank (PDB). The EMDB accession codes and PDB DOIs for all nucleosome-only controls, WGR-only complexes, PARP2–nucleosome complexes, PARP2–HPF1 complexes, and PARP2–HPF1 complexes bound to olaparib, EB-47, or talazoparib are listed in Supplementary Table S5. All other data supporting the findings of this study are available from the corresponding author upon reasonable request.

## Notes

Present Address: Program in Cellular and Molecular Medicine, Boston Children’s Hospital, Boston, MA 02115, United States
