# Supplementary material for: Structural basis of PARP2 recognition of nucleosomal DNA breaks and inhibitor trapping
Source: Nucleic Acids Res. 2026 Oct 5;54(18):gkag939. doi: 10.1093/nar/gkag939 (PMC13635751; doi:10.1093/nar/gkag939)
Supplement: Supplementary file 1 [file gkag939_supplemental_files.zip › Supp_nar-word-template_September4_2026_PDB_DOIs.docx]

**Supplementary Figure S1. Preparation and binding analysis of SSB-containing DNA and nucleosomes.** (**a**) Schematic of the 153-bp nucleosomal DNA containing a site-specific SSB at SHL +5.3, generating 130-nt and 23-nt fragments on the nicked strand. Representative gels show PCR-amplified DNA, nicking by denaturing gel electrophoresis, intact migration of the nicked DNA by native gel electrophoresis, and nucleosome reconstitution. (**b**) EMSA analysis of PARP2–HPF1 binding to free DNA containing an SSB at the SHL +5.3-equivalent position. Representative gel and quantification are shown, with an apparent *K_d_* of 653 ± 139 nM. (**c**) EMSA analysis of PARP2–HPF1 binding to free DNA without an SSB, showing minimal complex formation under the same conditions. (**d**) EMSA analysis of PARP2 binding to nucleosomes containing an SSB at SHL +5.3 in the absence or presence of HPF1. PARP2 binds the SSB-containing nucleosome with an apparent *K_d_* of 78.8 ± 5.1 nM, and addition of HPF1 produces a supershifted nucleosome–PARP2–HPF1 complex. (**e**) EMSAs were performed under the same conditions as in Fig. 1c, with a nucleosome containing an SSB at SHL +5.3 in the presence of 0.5 µM scFv, which was included to stabilize the nucleosome for structural studies. Increasing concentrations of PARP2 produced a pronounced mobility shift consistent with robust complex formation. Quantification of the bound fraction yielded a dissociation constant (*K_d_* = 74.4 ± 8.5 nM), comparable to that measured for nucleosomal SSB binding in the absence of scFv (Fig. 1c, left). This result indicates that scFv does not measurably affect PARP2 binding affinity.

**Supplementary Figure S2. NAD^+^- and HPF1-dependent PARP2 activity on nucleosomal and DNA SSB substrates.** (**a**) Alexa488 fluorescence gel analysis of H3 PARylation in reactions containing PARP2 with or without NAD^+^ and HPF1. PARylated H3 is detected only in the presence of both NAD⁺ and HPF1, whereas unmodified H3 remains near the bottom of the gel. (**b**) Coomassie-stained SDS–PAGE analysis of PARP2–HPF1 reactions with increasing NAD^+^ concentrations on nucleosomal or naked DNA substrates. Reactions were performed using nucleosomes containing an SSB at SHL +5.3 or intact nucleosomes, and the corresponding SSB-containing or intact DNA substrates. PARP2 and HPF1 PARylation is enhanced in the presence of SSB-containing nucleosome or SSB-containing DNA substrates compared with intact substrates. PARylated PARP2 and HPF1 species are indicated, along with unmodified PARP2, HPF1, and histones.

**Supplementary Figure. S3. Cryo-EM maps and validation of nucleosome-bound PARP2 complexes.** (**a**) Cryo-EM reconstruction of the PARP2–HPF1–scFv complex bound to an SSB-containing nucleosome, shown in four views related by 90° rotations. The map is colored by component: H2A, yellow; H2B, red; H3, blue; H4, green; DNA, pale colors; scFv, purple; PARP2, orange; HPF1, dark blue. (**b**) Local-resolution maps for the WGR-only, PARP2-only, and PARP2–HPF1 complexes bound to SSB-containing nucleosomes. Maps are shown in multiple orientations and colored according to local resolution. Zoomed-in views highlight density at the DNA break, including the SSB-binding region, and representative PARP2 features including R296 and the ART–HPF1 interface. (**c**) Gold-standard Fourier shell correlation (GSFSC) curves for local refinement and non-uniform refinement of the WGR, PARP2, and PARP2–HPF1 datasets. FSC curves are shown using different masking strategies, including no mask, spherical mask, loose mask, tight mask, and corrected FSC where applicable. Reported GSFSC resolutions are indicated above each plot.

**Supplementary Figure S4. Structural features of PARP2–HPF1 engagement with an SSB-containing nucleosome.** (**a**) Close-up view of the PARP2 WGR domain bound at the nucleosomal single-strand break, highlighting K183, Y201, and K130 near the damaged DNA terminus. (**b**) Additional PARP2–DNA contact mediated by R296 near the lower nucleosomal DNA gyre. (**c**) Close-up view of the HPF1–DNA interface, showing basic residues positioned near the lower nucleosomal DNA gyre, including K215, K211, R174, and K179. (**d**, **e**) Additional local contacts between PARP2 and nucleosomal DNA, involving Q159/W151 at the minor groove near the SSB and Q125/F126 between the two nucleosomal DNA gyres. (**f**) Structural comparison of the two binding mode 1 classes, Classes 1 (orange) and 2 (blue). Class 2 shows loss of ordered density C-terminal to G338, indicating increased flexibility or disorder of the downstream catalytic region relative to Class 1. The right panel shows a zoomed view of the helical domain near G338. (**g**, **h**) Structural comparison illustrating an open, NAD⁺-accessible ART-domain configuration observed in PARP2–HPF1 Class 2 (g) and a closed, NAD⁺-inaccessible ART-domain configuration observed in PARP2–HPF1 Class 1, which shows clear ART-domain density (h). The arrow indicates the direction of NAD⁺ access. (**i**) Per-nucleotide DNA RMSD comparing Class 3, binding mode 1, reference, with Class 5, binding mode 2. RMSDs are plotted separately for the SSB-containing strand and the complementary strand, highlighting enhanced deviations near the SSB. (**j**) Per-nucleotide DNA RMSD comparing Class 3, reference, with Class 8, binding mode 3, showing distinct DNA deformation patterns relative to binding mode 1.

**Supplementary Figure S5. Multiple sequence alignment of HPF1 highlights conservation of DNA-contacting basic residues.** Multiple sequence alignment of HPF1 orthologs from mammals, other vertebrates, and invertebrate animals. Residues corresponding to human HPF1 Arg174, Lys179, Lys211, and Lys215 are boxed. These basic residues form part of the HPF1 surface that contacts the lower gyre of nucleosomal DNA in the PARP2–HPF1–nucleosome structure. The conservation of these positions, particularly among vertebrate HPF1 orthologs, supports their functional importance in HPF1-mediated engagement of nucleosomal DNA. UniProt identifiers for each sequence are shown on the right.

**Supplementary Figure S6. Structural comparison of PARP2–HPF1 bound to nucleosomal DNA with other PARP2 structures.** (**a**) Superposition of the PARP2 WGR–DNA interface from the nucleosome-bound PARP2–HPF1 structure reported here with prior PARP2–DNA structures, 7AEO and 6USJ. The inset highlights conserved single-strand break–interacting residues, including K179, K183, and K130, as well as the nearby Y201 region, showing that PARP2 recognizes nucleosomal breaks through a DNA-binding mode similar to that observed on non-nucleosomal DNA. (**b**) Structural overlay of the WGR and helical domain regions shows that the nucleosome-bound PARP2 WGR–HD scaffold closely matches prior PARP2–DNA conformations, with only modest local differences near the WGR–HD junction. (**c**, **d**) Comparison of ART-domain positions after alignment on the WGR reveals substantial displacement of the catalytic ART domain in the nucleosome-bound complex relative to previously reported PARP2 conformations. Red dashed lines indicate distances between corresponding structural elements, highlighting large ART-domain repositioning relative to the DNA-bound scaffold. (**e**) Top, structural comparison of PARP2 bound to a nucleosomal SSB from this study (PARP2, orange; HPF1, blue) and the previously reported PARP2–HPF1 structure on linker DNA (PDB 6X0N; PARP2, green; HPF1, gray), shown in two orientations. Bottom, close-up view of the helical domain alignment between the two structures, showing relative shifts of helices αD, αE, and αF.

**Supplementary Figure S7. Cryo-EM analysis of nucleosomes containing an SSB at SHL +5.3 in complex with the PARP2 WGR domain.** (**a**) Superposition of the two cryo-EM structures determined from the WGR-only dataset, showing two WGR conformations relative to the SSB-containing nucleosome. Class 1 is shown in blue and Class 2 in orange. (**b**) Relative particle distribution of the two WGR conformational classes. Class 1 accounts for 51.0% of particles and Class 2 for 49.0%. (**c**) Cryo-EM maps and fitted models for Class 1 and Class 2, shown in the context of the nucleosome. Boxed regions are enlarged to show the WGR-domain density and model placement at the DNA break. The two classes show distinct WGR orientations relative to the nucleosome while maintaining engagement with the SSB site.

**Supplementary Figure S8. PARP inhibitors suppress PARP2–HPF1-dependent PARylation on SSB-containing nucleosomes.** *In vitro* PARylation assays were performed using the same PARP2, HPF1, and SSB-containing nucleosome constructs used for cryo-EM. Reactions contained 200 nM H3-Alexa Fluor 488-labeled SSB-containing nucleosomes, 1 µM PARP2, 3 µM HPF1, and 1 mM NAD^+^. Increasing concentrations of olaparib, talazoparib, or EB-47 were added at 0, 0.5, 1.0, 1.5, and 3.0 µM, as indicated by the triangles. Reactions were incubated at 37 °C for 25 min and resolved by SDS–PAGE. The A488 fluorescence scan (top) shows PARylated H3 species, while the Coomassie-stained gel (bottom) shows total PARP2 and HPF1, including slower-migrating PARylated PARP2 and HPF1 species. Increasing concentrations of each inhibitor reduce PARylation of H3, PARP2, and HPF1, confirming inhibitor binding under the conditions used for structural analysis.

**Supplementary Figure S9. PARP inhibitors differentially modulate PARP2–HPF1 conformational dynamics and nucleosomal DNA wrapping.** (**a**) Cryo-EM density maps and class distributions of nucleosomes in the absence of PARP inhibitors. Shown are conditions lacking PARP2 and HPF1 (left), containing PARP2 alone (middle), or containing both PARP2 and HPF1 (right). Representative maps are shown alongside pie charts summarizing the relative populations of fully wrapped and partially unwrapped nucleosomes, with the corresponding DNA wrap lengths indicated. (**b**) Cryo-EM classes obtained in the presence of talazoparib. Representative density maps for major and minor classes are shown, colored by class, with particle numbers, relative populations, map resolutions, and corresponding DNA wrap lengths indicated. The pie chart summarizes the distribution of nucleosome wrapping states across all talazoparib-bound classes. (**c**) Cryo-EM classes obtained in the presence of EB-47. Representative density maps and corresponding statistics are shown as in b, with a pie chart summarizing the relative populations of fully wrapped and partially unwrapped nucleosomes. (**d**) Conformational variability of the ART domain and HPF1 observed in inhibitor-bound datasets. Representative cryo-EM maps from talazoparib-bound (Classes 2 and 3) and EB-47-bound (Class 1) complexes are shown in two orthogonal views. Arrows indicate differences in the relative orientation of the ART domain and HPF1 across classes.

**Supplementary Figure S10. PARP inhibitors reduce WGR conformational heterogeneity and enrich WGR–HD–resolved states.** (**a**) Structural overlays of cryo-EM classes from the PARP2–nucleosome complex in the presence of talazoparib, grouped by binding modes and compared to the apo dataset. Binding mode 1 (Classes 1, 2, 3, and 7) shows minimal structural variability, whereas binding mode 2 (Classes 5, 6, and 8) displays lateral displacement along DNA, and binding mode 3 (Class 4) reflects motion toward or away from the nucleosomal DNA. Right, overlays of the helical domain highlighting inhibitor-dependent conformational changes in helix αF relative to apo. (**b**) Corresponding structural overlays for the EB-47 dataset. As observed for talazoparib and olaparib datasets, EB-47 reduces WGR conformational variability relative to apo and enriches classes in which both the WGR and helical domains are resolved. Notably, the C-terminal region of helix αF is resolved in this dataset, in contrast to the talazoparib and olaparib datasets. (**c**) Cryo-EM class population distributions for talazoparib, and EB-47. Top, outer rings group particles by binding mode, while inner rings show the relative population of individual classes. Bottom, distributions of classes resolving the WGR domain alone versus those resolving both the WGR and helical domains. Talazoparib and EB-47 markedly increase the population of WGR–HD–resolved states, biasing the ensemble toward conformations in which both the WGR and helical domains are resolved relative to the apo dataset.

**Supplementary Figure S11. Representative cryo-EM data processing workflow and resolution assessment.** (**a**) Representative processing workflow used for the WGR-only, PARP2-only, and PARP2–HPF1 datasets. Movies were subjected to patch motion correction, CTF estimation, and template-based particle picking, followed by 2D classification, heterogeneous refinement, and non-uniform refinement. Focused 3D classification using DNA entry- or exit-site masks separated bound, unbound, and partially unwrapped nucleosome populations. Selected particle classes were further classified with a ligand-focused mask, followed by reference-based motion correction, local CTF refinement, and local refinement. The final map is colored by local resolution. (**b**) Representative processing workflow used for PARP2–HPF1 datasets collected in the presence of PARP inhibitors, including EB-47, olaparib, and talazoparib. After initial motion correction, CTF estimation, particle picking, 2D classification, heterogeneous refinement, and non-uniform refinement, particles were separated by global and focused 3D classification using DNA entry-site, DNA exit-site, and ligand-focused masks. Bound, unbound, and unwrapped nucleosome classes were isolated, and selected classes were refined by reference-based motion correction, local CTF refinement, and local refinement. Final maps are colored by local resolution. Exact particle numbers for all datasets and classification steps are provided in Supplementary Table 3.

| DNA | Sequence (5’-3’) | Primers |
| --- | --- | --- |
| SHL +5.3 | ATCCTGGAGAATCCCGGTGCCGAGGCCGCTCAATTGGTCGTAGACAGCTCTAGCACCGCTTAAACGCACGTACGCGCTGTCCCCCGCGTTTTAACCGCCAAGGGGATTACTCCCTACTGACCAGGTCTCC**^**GTCAGATATATACATCCTGTGAT | FWD: 5’-Biotin-ATCCTGGAGAATCCCGGTGCCG-3’  RVS:5’-Biotin-ATCACAGGATGTATATATCTGACGGAG ACCTGGTC-3’ |

**Supplementary Table S1. Nucleosomal DNA and primer sequences.** DNA sequences (5′–3′) used to generate nucleosome substrates containing site-specific SSBs at SHL +5.3, together with the corresponding forward (FWD) and reverse (RVS) primers. The position of the engineered SSB is marked by a caret (^).

|  | Nucleosome with an SSB at SHL +5.3 (no PARP2) | Nucleosome-PARP2-HPF1, Apo | Nucleosome-PARP2 | Nucleosome-WGR domain | Nucleosome-PARP2-HPF1 with olaparib | Nucleosome-PARP2-HPF1 with talazoparib | Nucleosome-PARP2-HPF1 with EB-47 |
| --- | --- | --- | --- | --- | --- | --- | --- |
| Dose rate (e⁻/ Å^2^/s) | 18.9 | 17.3 | 17.5 | 23.2 | 18.7 | 25.4 | 19.9 |
| Total exposure (e⁻/Å^2^) | 45.4 | 44.9 | 45.5 | 44.5 | 48.5 | 49.4 | 50.0 |
| Exposure time (s) | 2.40 | 2.60 | 2.60 | 1.92 | 2.60 | 1.95 | 2.51 |
| Number of frames | 48 | 47 | 47 | 48 | 47 | 43 | 49 |

**Supplementary Table S2. Cryo-EM data acquisition parameters.** Summary of electron exposure conditions used for cryo-EM data collection across all datasets.

| **1. Initial acquisition and particle refinement** | | | | | | | |
| --- | --- | --- | --- | --- | --- | --- | --- |
| Complex | No PARP2 | WGR | PARP2 | PARP2-HPF1 | Olaparib | Talazo-parib | EB-47 |
| Movies | 8,046 | 12,970 | 8,205 | 7,734 | 6,905 | 8,818 | 7,512 |
| 2D class | 1,248,922 | 1,366,228 | 1,720,976 | 1,555,784 | 1,458,089 | 839,926 | 787,233 |
| Het Ref/NU Ref | 1,213,156 | 682,437 | 1,552,784 | 1,135,814 | 1,302,483 | 827,562 | 746,254 |
| **2. 3D classification without focus mask** | | | | | | | |
| Complex | No PARP2 | WGR | PARP2 | PARP2-HPF1 | Olaparib | Talazo-parib | EB-47 |
| PARP-**bound** particles per class in 1^st^ round 3D classification | N/A | 156,147  162,582 | 143,176  175,871  142,634  148,197  152,433  162,826  160,732  137,829  157,229 | 186,480  151,620  236,760  194,391  185,432  181,131 | 209,349  227,590  203,528  208,546  210,106 | 171,827  162,89  167,689  163,931  161,880 | 133,353  288,726  109,707  121,519 |
| **Unbound** particles in 1^st^ round 3D classification | N/A | 362,820 | 171,857 | 236,760 | 243,364 | 171,827 | 152,949 |
| **3. 3D classification with focus mask** | | | | | | | |
| Complex | No PARP2 | WGR | PARP2 | PARP2-HPF1 | Olaparib | Talazo-parib | EB-47 |
| PARP-**bound** particles per class in 2^nd^ round 3D classification | N/A | 156,147  162,582 | 58,957  65,386  50,170  66,521  75,253  65,723  71,633  56,043  103,710  156,495  162,130  151,669  71,844  70,630 | 26,256  75,535  90,585  184,658  97,876  95,854  89,855  82,433  82,433  74,593 | 122,382  133,222  139,762  92,725  86,760  47,071 | 235,477  27,024  30,675  25,517  65,900  70,411  101,394  63,023  24,999 | 132,787  109,233  186,819  70,034  40,739  22,661  26,630 |
| **Unbound** particles in 2^nd^ round 3D classification  (Drug complexes) | N/A | N/A | N/A | N/A | 124,344 | 139,524 | 113,061 |
| **4. 3D classification for unwrapping** | | | | | | | |
| Complex | No PARP2 | WGR | PARP2 | PARP2-HPF1 | Olaparib | Talazo-parib | EB-47 |
| Particles/percentage **unwrapped** from 3D classification (Non drug complexes) | 104,095  9% | 65,828  10% | 149,425  10% | 101,550  9% | N/A | N/A | N/A |
| Particles/ percentage ***unbound/ unwrapped*** in 3^nd^ round 3D classification  (Drug complexes) | N/A | N/A | N/A | N/A | 15,291  12% | 18,283  13% | 16,087  11% |

**Supplementary Table S3.** **Particle numbers associated with the cryo-EM data processing workflows shown in Supplementary Figure 11.** Particle counts are provided for each dataset at successive stages of image processing, including initial particle selection after heterogeneous and non-uniform refinement, first-round 3D classification without focused masks, second-round focused 3D classification, and classification of unwrapped nucleosome populations where applicable. Values are reported separately for each structural class, including PARP-bound, unbound, and unwrapped particles, for the WGR-only, PARP2-only, PARP2–HPF1, and PARP inhibitor–bound (olaparib, talazoparib, and EB-47) datasets. For cells containing multiple particle counts, values are listed from top to bottom in sequential class order, beginning with Class 1, followed by Class 2, Class 3, and subsequent classes.

**Supplementary Table S4.**  **Cryo-EM data collection and model refinement statistics.** **(separate file)**

Cryo-EM acquisition, reconstruction, and atomic model validation parameters for nucleosome, WGR-only, PARP2-only, and PARP2–HPF1 complexes in the absence or presence of PARP inhibitors (olaparib, EB-47, talazoparib). For datasets with multiple classes, statistics are reported per class with corresponding PDB and EMDB accession codes. Map resolutions are reported at FSC = 0.143. Model quality metrics include r.m.s. deviations, MolProbity scores, clash scores, rotamer statistics, and Ramachandran analysis.

| **Without PARP2** | | | | | | | | |
| --- | --- | --- | --- | --- | --- | --- | --- | --- |
| **Class** | **Type** | | | **PDB DOI** | | **Extended PDB** | | **EMDB** |
| 1 | Main | | | 10.2210/pdb9ylr/pdb | | PDB_0000PYLR | | EMD-73092 |
| **WGR** | | | | | | | | |
| **Class** | **Type** | | | **PDB DOI** | | **Extended PDB** | | **EMDB** |
| 1 | Main/Composite | | | 10.2210/pdbptun/pdb | | PDB_0000PTUN | | EMD-56267 |
| 1 | Consensus | | |  | |  | | EMD-75017 |
| 1 | Focus | | |  | |  | | EMD-75018 |
| 2 | Main/Composite | | | 10.2210/pdbpzzz/pdb | | PDB_00009ZZZ | | EMD-75022 |
| 2 | Consensus | | |  | |  | | EMD-75019 |
| 2 | Focus | | |  | |  | | EMD-75021 |
| **PARP2** | | | | | | | | |
| **Class** | **Type** | | | **PDB DOI** | | **Extended PDB** | | **EMDB** |
| 1 | Main/Composite | | | 10.2210/pdb9zta/pdb | | PDB_00009ZTA | | EMD-74745 |
| 1 | Consensus | | |  | |  | | EMD-74703 |
| 1 | Focus | | |  | |  | | EMD-74704 |
| 2 | Main/Composite | | | 10.2210/pdb9ztf/pdb | | PDB_00009ZTF | | EMD-74751 |
| 2 | Consensus | | |  | |  | | EMD-74705 |
| 2 | Focus | | |  | |  | | EMD-74707 |
| 3 | Main/Composite | | | 10.2210/pdb9ztg/pdb | | PDB_00009ZTG | | EMD-74752 |
| 3 | Consensus | | |  | |  | | EMD-74709 |
| 3 | Focus | | |  | |  | | EMD-74710 |
| 4 | Main/Composite | | | 10.2210/pdb9zth/pdb | | PDB_00009ZTH | | EMD-74753 |
| 4 | Consensus | | |  | |  | | EMD-74714 |
| 4 | Focus | | |  | |  | | EMD-74715 |
| 5 | Main/Composite | | | 10.2210/pdb9ztq/pdb | | PDB_00009ZTQ | | EMD-74769 |
| 5 | Consensus | | |  | |  | | EMD-74722 |
| 5 | Focus | | |  | |  | | EMD-74760 |
| 6 | Main/Composite | | | 10.2210/pdb9zti/pdb | | PDB_00009ZTI | | EMD-74754 |
| 6 | Consensus | | |  | |  | | NA |
| 6 | Focus | | |  | |  | | NA |
| 7 | Main/Composite | | | 10.2210/pdb9zu5/pdb | | PDB_00009ZU5 | | EMD-74793 |
| 7 | Consensus | | |  | |  | | EMD-74747 |
| 7 | Focus | | |  | |  | | EMD-74748 |
| 8 | Main/Composite | | | 10.2210/pdb9zy2/pdb | | PDB_00009ZY2 | | EMD-74941 |
| 8 | Consensus | | |  | |  | | EMD-74758 |
| 8 | Focus | | |  | |  | | EMD-74759 |
| 9 | Main/Composite | | | 10.2210/pdb9ztm/pdb | | PDB_00009ZTM | | EMD-74764 |
| 9 | Consensus | | |  | |  | | EMD-74761 |
| 9 | Focus | | |  | |  | | EMD-74762 |
| 10 | Main/Composite | | | 10.2210/pdb9zu0/pdb | | PDB_00009ZU0 | | EMD-74785 |
| 10 | Consensus | | |  | |  | | EMD-74782 |
| 10 | Focus | | |  | |  | | EMD-74780 |
| 11 | Main/Composite | | | 10.2210/pdb9zu8/pdb | | PDB_00009ZU8 | | EMD-74796 |
| 11 | Consensus | | |  | |  | | EMD-74790 |
| 11 | Focus | | |  | |  | | EMD-74791 |
| 12 | Main/Composite | | | 10.2210/pdb9zu7/pdb | | PDB_00009ZU7 | | EMD-74795 |
| 12 | Consensus | | |  | |  | | NA |
| 12 | Focus | | |  | |  | | NA |
| 13 | Main/Composite | | | 10.2210/pdbpzua/pdb | | PDB_0000PZUA | | EMD-74800 |
| 13 | Consensus | | |  | |  | | NA |
| 13 | Focus | | |  | |  | | NA |
| 14 | Main/Composite | | | 10.2210/pdb9zxl/pdb | | PDB_00009ZXL | | EMD-74928 |
| 14 | Consensus | | |  | |  | | EMD-74803 |
| 14 | Focus | | |  | |  | | EMD-74804 |
| **PARP2_HPF1** | | | | | | | | |
| **Class** | **Type** | | | **PDB DOI** | | **Extended PDB** | | **EMDB** |
| 1 | Main/Composite | | | 10.2210/pdb9ztn/pdb | | PDB_00009ZTN | | EMD-74766 |
| 1 | Consensus | | |  | |  | | EMD-74590 |
| 1 | Focus | | |  | |  | | EMD-74593 |
| 2 | Main/Composite | | | 10.2210/pdb9zto/pdb | | PDB_00009ZTO | | EMD-74767 |
| 2 | Consensus | | |  | |  | | EMD-74595 |
| 2 | Focus | | |  | |  | | EMD-74598 |
| 3 | Main/Composite | | | 10.2210/pdb9ztp/pdb | | PDB_00009ZTP | | EMD-74768 |
| 3 | Consensus | | |  | |  | | EMD-74604 |
| 3 | Focus | | |  | |  | | EMD-74606 |
| 4 | Main/Composite | | | 10.2210/pdb9ztr/pdb | | PDB_00009ZTR | | EMD-74770 |
| 4 | Consensus | | |  | |  | | NA |
| 4 | Focus | | |  | |  | | NA |
| 5 | Main/Composite | | | 10.2210/pdb9zts/pdb | | PDB_00009ZTS | | EMD-74771 |
| 5 | Consensus | | |  | |  | | NA |
| 5 | Focus | | |  | |  | | NA |
| 6 | Main/Composite | | | 10.2210/pdb9ztu/pdb | | PDB_00009ZTU | | EMD-74773 |
| 6 | Consensus | | |  | |  | | EMD-74607 |
| 6 | Focus | | |  | |  | | EMD-74611 |
| 7 | Main/Composite | | | 10.2210/pdb9ztw/pdb | | PDB_00009ZTW | | EMD-74775 |
| 7 | Consensus | | |  | |  | | EMD-74612 |
| 7 | Focus | | |  | |  | | EMD-74613 |
| 8 | Main/Composite | | | 10.2210/pdb9ztx/pdb | | PDB_00009ZTX | | EMD-74776 |
| 8 | Consensus | | |  | |  | | NA |
| 8 | Focus | | |  | |  | | NA |
| 9 | Main/Composite | | | 10.2210/pdb9ztz/pdb | | PDB_00009ZTZ | | EMD-74784 |
| 9 | Consensus | | |  | |  | | NA |
| 9 | Focus | | |  | |  | | NA |
| 10 | Main/Composite | | | 10.2210/pdb9zty/pdb | | PDB_00009ZTY | | EMD-74778 |
| 10 | Consensus | | |  | |  | | NA |
| 10 | Focus | | |  | |  | | NA |
| **PARP2_HPF1+Olaparib** | | | | | | | | |
| **Class** | | **Type** | **PDB DOI** | | **Extended PDB** | | **EMDB** | |
| 1 | | Main/Composite | 10.2210/pdb9zyc/pdb | | PDB_00009ZYC | | EMD-74947 | |
| 1 | | Consensus |  | |  | | EMD-74689 | |
| 1 | | Focus |  | |  | | EMD-74690 | |
| 2 | | Main/Composite | 10.2210/pdb9zsu/pdb | | PDB_00009ZSU | | EMD-74727 | |
| 2 | | Consensus |  | |  | | EMD-74671 | |
| 2 | | Focus |  | |  | | EMD-74672 | |
| 3 | | Main/Composite | 10.2210/pdb9zsw/pdb | | PDB_00009ZSW | | EMD-74728 | |
| 3 | | Consensus |  | |  | | EMD-74673 | |
| 3 | | Focus |  | |  | | EMD-74674 | |
| 4 | | Main/Composite | 10.2210/pdb9zsx/pdb | | PDB_00009ZSX; | | EMD-74729 | |
| 4 | | Consensus |  | |  | | EMD-74676 | |
| 4 | | Focus |  | |  | | EMD-74677 | |
| 5 | | Main/Composite | 10.2210/pdb9zsy/pdb | | PDB_00009ZSY | | EMD-74730 | |
| 5 | | Consensus |  | |  | | EMD-74679 | |
| 5 | | Focus |  | |  | | EMD-74682 | |
| 6 | | Main/Composite | 10.2210/pdb9zsz/pdb | | PDB_00009ZSZ | | EMD-74731 | |
| 6 | | Consensus |  | |  | | EMD-74686 | |
| 6 | | Focus |  | |  | | EMD-74687 | |
| **PARP2_HPF1+EB-47** | | | | | | | | |
| **Class** | | **Type** | **PDB DOI** | | **Extended PDB** | | **EMDB** | |
| 1 | | Main/Composite | 10.2210/pdb9zte/pdb | | PDB_00009ZTE | | EMD-74750 | |
| 1 | | Consensus |  | |  | | NA | |
| 1 | | Focus |  | |  | | NA | |
| 2 | | Main/Composite | 10.2210/pdb9zsp/pdb | | PDB_00009ZSP | | EMD-74721 | |
| 2 | | Consensus |  | |  | | EMD-74649 | |
| 2 | | Focus |  | |  | | EMD-74653 | |
| 3 | | Main/Composite | 10.2210/pdb9zsq/pdb | | PDB_00009ZSQ | | EMD-74723 | |
| 3 | | Consensus |  | |  | | EMD-74651 | |
| 3 | | Focus |  | |  | | EMD-74652 | |
| 4 | | Main/Composite | 10.2210/pdb9zsr/pdb | | PDB_00009ZSR | | EMD-74724 | |
| 4 | | Consensus |  | |  | | EMD-74659 | |
| 4 | | Focus |  | |  | | EMD-74660 | |
| 5 | | Main/Composite | 10.2210/pdb9ztd/pdb | | PDB_00009ZTD | | EMD-74749 | |
| 5 | | Consensus |  | |  | | NA | |
| 5 | | Focus |  | |  | | NA | |
| 6 | | Main/Composite | 10.2210/pdb9zss/pdb | | PDB_00009ZSS | | EMD-74725 | |
| 6 | | Consensus |  | |  | | EMD-74661 | |
| 6 | | Focus |  | |  | | EMD-74663 | |
| 7 | | Main/Composite | 10.2210/pdb9zst/pdb | | PDB_00009ZST | | EMD-74726 | |
| 7 | | Consensus |  | |  | | EMD-74662 | |
| 7 | | Focus |  | |  | | EMD-74669 | |
| **PARP2_HPF1+Talazoparib** | | | | | | | | |
| **Class** | | **Type** | **PDB DOI** | | **Extended PDB** | | **EMDB** | |
| 1 | | Main/Composite | 10.2210/pdb9zs5/pdb | | PDB_00009ZS5 | | EMD-74691 | |
| 1 | | Consensus |  | |  | | EMD-74597 | |
| 1 | | Focus |  | |  | | EMD-74600 | |
| 2 | | Main/Composite | 10.2210/pdb9zs6/pdb | | PDB_00009ZS6 | | EMD-74693 | |
| 2 | | Consensus |  | |  | | EMD-74601 | |
| 2 | | Focus |  | |  | | EMD-74602 | |
| 3 | | Main/Composite | 10.2210/pdb9zsg/pdb | | PDB_00009ZSG; | | EMD-74711 | |
| 3 | | Consensus |  | |  | | EMD-74603 | |
| 3 | | Focus |  | |  | | EMD-74605 | |
| 4 | | Main/Composite | 10.2210/pdb9zsi/pdb | | PDB_00009ZSI | | EMD-74712 | |
| 4 | | Consensus |  | |  | | EMD-74609 | |
| 4 | | Focus |  | |  | | EMD-74610 | |
| 5 | | Main/Composite | 10.2210/pdb9zsj/pdb | | PDB_00009ZSJ | | EMD-74713 | |
| 5 | | Consensus |  | |  | | EMD-74631 | |
| 5 | | Focus |  | |  | | EMD-74632 | |
| 6 | | Main/Composite | 10.2210/pdb9zsk/pdb | | PDB_00009ZSK | | EMD-74716 | |
| 6 | | Consensus |  | |  | | EMD-74633 | |
| 6 | | Focus |  | |  | | EMD-74634 | |
| 7 | | Main/Composite | 10.2210/pdb9zsl/pdb | | PDB_00009ZSL | | EMD-74717 | |
| 7 | | Consensus |  | |  | | EMD-74647 | |
| 7 | | Focus |  | |  | | EMD-74648 | |
| 8 | | Main/Composite | 10.2210/pdb9zsm/pdb | | PDB_00009ZSM; | | EMD-74719 | |
| 8 | | Consensus |  | |  | | EMD-74635 | |
| 8 | | Focus |  | |  | | EMD-74636 | |
| 9 | | Main/Composite | 10.2210/pdb9zso/pdb | | PDB_00009ZSO | | EMD-74720 | |
| 9 | | Consensus |  | |  | | EMD-74637 | |
| 9 | | Focus |  | |  | | EMD-74638 | |

**Supplementary Table S5. Cryo-EM Map and Model Depositions for PARP2–Nucleosome Complexes.** Summary of cryo-EM reconstructions and corresponding PDB and EMDB depositions for nucleosome-only controls and PARP2-containing complexes, including WGR-only, PARP2, PARP2–HPF1, and PARP2–HPF1 bound to olaparib, EB-47, or talazoparib. For each dataset, class-specific main/composite maps and, where available, consensus and focused reconstructions are listed with their associated accession codes. Entries marked “NA” for consensus or focused maps indicate cases in which the deposition is not a composite reconstruction.

**Supplementary Movie S1. PARP2–HPF1 binding distorts the DNA backbone at the single-strand break.** Morph between the nucleosome structure containing an SSB at SHL +5.3 in the absence of PARP2–HPF1 and the PARP2–HPF1-bound structure. The movie illustrates repositioning of the DNA backbone at the break site upon PARP2–HPF1 binding.

**Supplementary Movie S2. Lateral repositioning of the PARP2 WGR domain and nucleosomal DNA in binding mode 2.** Morph between representative classes in binding mode 2. The movie illustrates lateral movement of the PARP2 WGR domain along the nucleosomal DNA, accompanied by concerted displacement of the DNA.

**Supplementary Movie S3. WGR-domain helix movement relative to nucleosomal DNA in binding mode 3.** Morph between representative classes in binding mode 3. The movie illustrates movement of a WGR-domain helix toward or away from the nucleosomal DNA, highlighting conformational variability within the DNA-bound WGR domain.
