# Supplementary material for: Structural basis of PARP2 recognition of nucleosomal DNA breaks and inhibitor trapping
Source: Nucleic Acids Res. 2026 Oct 5;54(18):gkag939. doi: 10.1093/nar/gkag939 (PMC13635751; doi:10.1093/nar/gkag939)
Supplement: Supplementary file 1 [file gkag939_supplemental_files.zip › Supp_Table_4_July6_2026.docx]

| **Nucleosome with an SSB at SHL +5.3** | |
| --- | --- |
| PDB ID | 9YLR |
| EMDB ID | EMD-73092 |
| *Data collection and processing* | |
| Magnification | 105,000 |
| Voltage (kV) | 300 |
| Electron exposure (e–/Å^2^) | 45 |
| Defocus range (μm) | -1.8 to -0.8 |
| Pixel size (Å) | 0.414 |
| Movies collected | 8,046 |
| Symmetry imposed | C1 |
| FSC threshold | 0.143 |
| Initial particle images (no.) | 1,217,466 |
| Final particle images (no.) | 1,213,156 |
| Map resolution (Å) | 1.99 |
| Model resolution (Å) | 1.90 |
| *Model composition* | |
| Non-hydrogen atoms | 16,018 |
| Protein residues | 1232 |
| DNA bases | 306 |
| *R.m.s deviations* | |
| Bond lengths (Å) | 0.006 |
| Bond angles (°) | 0.907 |
| MolProbity score | 1.12 |
| Clashscore | 3.09 |
| Poor rotamers (%) | 0.86 |
| *Ramachandran plot* | |
| Favored (%) | 97.93 |
| Allowed (%) | 2.07 |
| Outliers (%) | 0 |

| **Nucleosome with an SSB at SHL +5.3 bound to the PARP2 WGR domain** | | |
| --- | --- | --- |
| Class | 1 | 2 |
| PDB ID | 9TUN | 9ZZZ |
| EMDB ID | EMD-56267 | EMD-75022 |
| *Data collection and processing* | | |
| Magnification | 105,000 | 105,000 |
| Voltage (kV) | 300 | 300 |
| Electron exposure (e–/Å^2^) | 45 | 45 |
| Defocus range (μm) | -1.8 to -0.8 | -1.8 to -0.8 |
| Pixel size (Å) | 0.414 | 0.414 |
| Movies collected | 12,970 | 12,970 |
| Symmetry imposed | C1 | C1 |
| FSC threshold | 0.143 | 0.143 |
| Initial particle images (no.) | 682,437 | 682,437 |
| Final particle images (no.) | 162,528 | 156,147 |
| Map resolution (Å) | 2.59 | 2.56 |
| Model resolution (Å) | 2.24 | 2.24 |
| *Model composition* | | |
| Non-hydrogen atoms | 16,947 | 16,938 |
| Protein residues | 1345 | 1344 |
| DNA bases | 306 | 306 |
| *R.m.s. deviations* | | |
| Bond lengths (Å) | 0.005 | 0.006 |
| Bond angles (°) | 0.866 | 0.885 |
| MolProbity score | 1.70 | 1.77 |
| Clashscore | 5.44 | 6.53 |
| Poor rotamers (%) | 1.48 | 1.40 |
| *Ramachandran plot* | | |
| Favored (%) | 95.98 | 95.68 |
| Allowed (%) | 3.94 | 4.25 |
| Outliers (%) | 0.08 | 0.08 |

| **Nucleosome with an SSB at SHL +5.3 bound to PARP2 in the absence of HPF1** | | | | | | | | | | | | | | |
| --- | --- | --- | --- | --- | --- | --- | --- | --- | --- | --- | --- | --- | --- | --- |
| Class | 1 | 2 | 3 | 4 | 5 | 6 | 7 | 8 | 9 | 10 | 11 | 12 | 13 | 14 |
| PDB ID | 9ZTA | 9ZTF | 9ZTG | 9ZTH | 9ZTQ | 9ZTI | 9ZU5 | 9ZY2 | 9ZTM | 9ZU0 | 9ZU8 | 9ZU7 | PZUA | 9ZXL |
| EMDB ID | EMD-74745 | EMD-74751 | EMD-74752 | EMD-74753 | EMD-74769 | EMD-74754 | EMD-74793 | EMD-74941 | EMD-74764 | EMD-74785 | EMD-74796 | EMD-74795 | EMD-74800 | EMD-74928 |
| *Data collection and processing* | | | | | | | | | | | | | | |
| Magnification | 105,000 | 105,000 | 105000 | 105,000 | 105,000 | 105,000 | 105,000 | 105,000 | 105,000 | 105,000 | 105,000 | 105,000 | 105,000 | 105,000 |
| Voltage (kV) | 300 | 300 | 300 | 300 | 300 | 300 | 300 | 300 | 300 | 300 | 300 | 300 | 300 | 300 |
| Electron exposure (e–/Å^2^) | 45 | 45 | 45 | 45 | 45 | 45 | 45 | 45 | 45 | 45 | 45 | 45 | 45 | 45 |
| Defocus range (μm) | -1.8 to -0.8 | -1.8 to -0.8 | -1.8 to -0.8 | -1.8 to -0.8 | -1.8 to -0.8 | -1.8 to -0.8 | -1.8 to -0.8 | -1.8 to -0.8 | -1.8 to -0.8 | -1.8 to -0.8 | -1.8 to -0.8 | -1.8 to -0.8 | -1.8 to -0.8 | -1.8 to -0.8 |
| Pixel size (Å) | 0.414 | 0.414 | 0.414 | 0.414 | 0.414 | 0.414 | 0.414 | 0.414 | 0.414 | 0.414 | 0.414 | 0.414 | 0.414 | 0.414 |
| Movies collected | 8,205 | 8,205 | 8,205 | 8,205 | 8,205 | 8,205 | 8,205 | 8,205 | 8,205 | 8,205 | 8,205 | 8,205 | 8,205 | 8,205 |
| Symmetry imposed | C1 | C1 | C1 | C1 | C1 | C1 | C1 | C1 | C1 | C1 | C1 | C1 | C1 | C1 |
| FSC threshold | 0.143 | 0.143 | 0.143 | 0.143 | 0.143 | 0.143 | 0.143 | 0.143 | 0.143 | 0.143 | 0.143 | 0.143 | 0.143 | 0.143 |
| Initial particle images (no.) | 1,552,784 | 1,552,784 | 1,552,784 | 1,552,784 | 1,552,784 | 1,552,784 | 1,552,784 | 1,552,784 | 1,552,784 | 1,552,784 | 1,552,784 | 1,552,784 | 1,552,784 | 1,552,784 |
| Final particle images (no.) | 58,957 | 65,386 | 50,170 | 66,521 | 75,253 | 65,723 | 81,633 | 56,043 | 103,710 | 156,495 | 162,130 | 151,669 | 71,844 | 70,630 |
| Map resolution (Å) | 2.65 | 2.66 | 2.68 | 2.64 | 2.63 | 2.57 | 2.55 | 2.59 | 2.49 | 2.33 | 2.33 | 2.39 | 2.53 | 2.48 |
| Model resolution (Å) | 2.19 | 2.05 | 2.07 | 2.15 | 2.23 | 2.57 | 2.12 | 2.10 | 2.04 | 2.00 | 2.07 | 2.38 | 2.53 | 2.07 |
| *Model composition* | | | | | | | | | | | | | | |
| Non-hydrogen atoms | 17576 | 17204 | 17642 | 16966 | 17502 | 16898 | 16951 | 17642 | 16951 | 16951 | 17420 | 16935 | 16951 | 17560 |
| Protein residues | 1425 | 1377 | 1432 | 1348 | 1415 | 1339 | 1345 | 1432 | 1345 | 1345 | 1406 | 1432 | 1345 | 1422 |
| DNA bases | 306 | 306 | 306 | 306 | 306 | 306 | 306 | 306 | 306 | 306 | 306 | 306 | 306 | 306 |
| *R.m.s. deviations* | | | | | | | | | | | | | | |
| Bond lengths (Å) | 0.006 | 0.004 | 0.005 | 0.005 | 0.004 | 0.004 | 0.005 | 0.006 | 0.005 | 0.005 | 0.005 | 0.005 | 0.006 | 0.005 |
| Bond angles (°) | 0.863 | 0.840 | 0.870 | 0.853 | 0.868 | 0.866 | 0.875 | 0.892 | 0.657 | 0.870 | 0.882 | 0.862 | 0.871 | 0.887 |
| MolProbity score | 1.67 | 1.44 | 1.24 | 1.16 | 1.83 | 1.21 | 1.63 | 1.66 | 1.10 | 1.25 | 1.26 | 1.14 | 1.20 | 1.84 |
| Clashscore | 4.61 | 3.12 | 3.82 | 3.13 | 5.68 | 4.14 | 5.37 | 6.02 | 2.44 | 3.96 | 3.85 | 3.52 | 3.52 | 6.33 |
| Poor rotamers (%) | 1.98 | 1.88 | 1.23 | 0.70 | 2.07 | 0.70 | 1.48 | 1.39 | 0.79 | 1.22 | 0.75 | 0.79 | 1.05 | 1.82 |
| *Ramachandran plot* | | | | | | | | | | |  |  |  |  |
| Favored (%) | 96.64 | 97.33 | 98.01 | 97.73 | 95.96 | 97.94 | 96.66 | 96.58 | 97.65 | 98.03 | 97.60 | 98.55 | 97.80 | 95.70 |
| Allowed (%) | 3.36 | 2.67 | 1.92 | 2.27 | 3.83 | 2.06 | 3.26 | 3.42 | 2.27 | 1.97 | 2.40 | 1.45 | 2.20 | *4.23* |
| Outliers (%) | 0 | 0 | 0.07 | 0 | 0.21 | 0 | 0.08 | 0 | 0.08 | 0 | 0 | 0 | 0 | 0.07 |

| **Nucleosome with an SSB at SHL +5.3 bound to PARP2-HPF1** | | | | | | | | | | |
| --- | --- | --- | --- | --- | --- | --- | --- | --- | --- | --- |
| Class | 1 | 2 | 3 | 4 | 5 | 6 | 7 | 8 | 9 | 10 |
| PDB ID | 9ZTN | 9ZTO | 9ZTP | 9ZTR | 9ZTS | 9ZTU | 9ZTW | 9ZTX | 9ZTZ | 9ZTY |
| EMDB ID | EMD-74766 | EMD-74767 | EMD-74768 | EMD-74770 | EMD-74771 | EMD-74773 | EMD-74775 | EMD-74776 | EMD-74784 | EMD-74778 |
| *Data collection and processing* | | | | | | | | | | |
| Magnification | 105,000 | 105,000 | 105,000 | 105,000 | 105,000 | 105,000 | 105,000 | 105,000 | 105,000 | 105,000 |
| Voltage (kV) | 300 | 300 | 300 | 300 | 300 | 300 | 300 | 300 | 300 | 300 |
| Electron exposure (e–/Å^2^) | 45 | 45 | 45 | 45 | 45 | 45 | 45 | 45 | 45 | 45 |
| Defocus range (μm) | -1.8 to -0.8 | -1.8 to -0.8 | -1.8 to -0.8 | -1.8 to -0.8 | -1.8 to -0.8 | -1.8 to -0.8 | -1.8 to -0.8 | -1.8 to -0.8 | -1.8 to -0.8 | -1.8 to -0.8 |
| Pixel size (Å) | 0.414 | 0.414 | 0.414 | 0.414 | 0.414 | 0.414 | 0.414 | 0.414 | 0.414 | 0.414 |
| Movies collected | 7,734 | 7,734 | 7,734 | 7,734 | 7,734 | 7,734 | 7,734 | 7,734 | 7,734 | 7,734 |
| Symmetry imposed | C1 | C1 | C1 | C1 | C1 | C1 | C1 | C1 | C1 | C1 |
| FSC threshold | 0.143 | 0.143 | 0.143 | 0.143 | 0.143 | 0.143 | 0.143 | 0.143 | 0.143 | 0.143 |
| Initial particle images (no.) | 1,135,814 | 1,135,814 | 1,135,814 | 1,135,814 | 1,135,814 | 1,135,814 | 1,135,814 | 1,135,814 | 1,135,814 | 1,135,814 |
| Final particle images (no.) | 26,256 | 75,535 | 90,585 | 184,658 | 97,876 | 95,854 | 89,855 | 82,433 | 82,433 | 74,593 |
| Map resolution (Å) | 2.84 | 3.14 | 2.49 | 2.33 | 2.50 | 2.48 | 2.51 | 2.51 | 2.49 | 2.58 |
| Model resolution (Å) | 2.56 | 1.61 | 1.63 | 2.15 | 2.48 | 1.61 | 1.62 | 2.49 | 2.50 | 2.57 |
| *Model composition* | | | | | | | | | | |
| Non-hydrogen atoms | 22,355 | 17,564 | 16,971 | 16,971 | 16,737 | 16,969 | 16,969 | 16,648 | 16,945 | 16,969 |
| Protein residues | 2019 | 1424 | 1348 | 1348 | 1318 | 1348 | 1348 | 1307 | 1344 | 1348 |
| DNA bases | 306 | 306 | 306 | 306 | 306 | 306 | 306 | 306 | 306 | 306 |
| *R.m.s deviations* | | | | | | | | | | |
| Bond lengths (Å) | 0.006 | 0.006 | 0.006 | 0.005 | 0.006 | 0.005 | 0.006 | 0.004 | 0.005 | 0.005 |
| Bond angles (°) | 0.880 | 0.865 | 0.890 | 0.861 | 0.899 | 0.856 | 0.875 | 0.843 | 0.847 | 0.856 |
| MolProbity score | 1.40 | 1.50 | 1.45 | 1.30 | 1.22 | 1.23 | 1.43 | 1.19 | 1.34 | 1.53 |
| Clashscore | 3.75 | 3.44 | 3.06 | 2.71 | 4.40 | 3.99 | 3.99 | 3.52 | 4.15 | 5.17 |
| Poor rotamers (%) | 1.04 | 1.98 | 1.39 | 1.74 | 0.45 | 1.13 | 1.39 | 1.17 | 1.13 | 1.22 |
| *Ramachandran plot* | | | | | | | | | | |
| Favored (%) | 96.58 | 97.28 | 96.37 | 97.73 | 98.21 | 98.11 | 97.28 | 98.43 | 97.49 | 96.82 |
| Allowed (%) | 3.37 | 2.72 | 3.63 | 2.27 | 1.79 | 1.89 | 2.72 | 1.57 | 2.51 | 3.18 |
| Outliers (%) | 0.05 | 0 | 0 | 0 | 0 | 0 | 0 | 0 | 0 | 0 |

| **Nucleosome with an SSB at SHL +5.3 bound to PARP2–HPF1 in the presence of olaparib** | | | | | | |
| --- | --- | --- | --- | --- | --- | --- |
| Class | 1 | 2 | 3 | 4 | 5 | 6 |
| PDB ID | 9ZYC | 9ZSU | 9ZSW | 9ZSX | 9ZSY | 9ZSZ |
| EMDB ID | EMD-74947 | EMD-74727 | EMD-74728 | EMD-74729 | EMD-74730 | EMD-74731 |
| *Data collection and processing* | | | | | | |
| Magnification | 105,000 | 105,000 | 105,000 | 105,000 | 105,000 | 105,000 |
| Voltage (kV) | 300 | 300 | 300 | 300 | 300 | 300 |
| Electron exposure (e–/Å^2^) | 48 | 48 | 48 | 48 | 48 | 48 |
| Defocus range (μm) | -1.8 to -0.8 | -1.8 to -0.8 | -1.8 to -0.8 | -1.8 to -0.8 | -1.8 to -0.8 | -1.8 to -0.8 |
| Pixel size (Å) | 0.414 | 0.414 | 0.414 | 0.414 | 0.414 | 0.414 |
| Movies collected | 6,095 | 6,095 | 6,095 | 6,095 | 6,095 | 6,095 |
| Symmetry imposed | C1 | C1 | C1 | C1 | C1 | C1 |
| FSC threshold | 0.143 | 0.143 | 0.143 | 0.143 | 0.143 | 0.143 |
| Initial particle images (no.) | 1,302,483 | 1,302,483 | 1,302,483 | 1,302,483 | 1,302,483 | 1,302,483 |
| Final particle images (no.) | 122,382 | 133,222 | 139,762 | 92,725 | 86,760 | 47,071 |
| Map resolution (Å) | 2.26 | 2.21 | 2.21 | 2.34 | 2.37 | 2.46 |
| Model resolution (Å) | 1.90 | 1.83 | 1.65 | 2.02 | 2.02 | 1.95 |
| *Model composition* | | | | | | |
| Non-hydrogen atoms | 17,635 | 17,646 | 17,605 | 16,657 | 16,771 | 14,955 |
| Protein residues | 1431 | 1433 | 1428 | 1350 | 1349 | 1227 |
| DNA bases | 306 | 306 | 306 | 290 | 296 | 256 |
| *R.m.s deviations* | | | | | | |
| Bond lengths (Å) | 0.005 | 0.005 | 0.005 | 0.005 | 0.004 | 0.005 |
| Bond angles (°) | 0.641 | 0.840 | 0.859 | 0.868 | 0.845 | 0.862 |
| MolProbity score | 1.45 | 1.43 | 1.40 | 1.41 | 1.34 | 1.06 |
| Clashscore | 4.99 | 4.92 | 3.89 | 4.05 | 4.10 | 2.74 |
| Poor rotamers (%) | 1.23 | 1.47 | 1.56 | 1.39 | 0.87 | 0.77 |
| *Ramachandran plot* | | | | | | |
| Favored (%) | 97.36 | 97.79 | 97.64 | 97.43 | 97.20 | 98.00 |
| Allowed (%) | 2.57 | 2.14 | 2.29 | 2.57 | 2.72 | 1.91 |
| Outliers (%) | 0.07 | 0.07 | 0.07 | 0 | 0.08 | 0.08 |

| **Nucleosome with an SSB at SHL +5.3 bound to PARP2–HPF1 in the presence of EB-47** | | | | | | | |
| --- | --- | --- | --- | --- | --- | --- | --- |
| Class | Class 1 | Class 2 | Class 3 | Class 4 | Class 5 | Class 6 | Class 7 |
| PDB ID | 9ZTE | 9ZSP | 9ZSQ | 9ZSR | 9ZTD | 9ZSS | 9ZST |
| EMDB ID | EMD-74750 | EMD-74721 | EMD-74723 | EMD-74724 | EMD-74749 | EMD-74725 | EMD-74726 |
| *Data collection and processing* | | | | | | | |
| Magnification | 105,000 | 105,000 | 105,000 | 105,000 | 105,000 | 105,000 | 105,000 |
| Voltage (kV) | 300 | 300 | 300 | 300 | 300 | 300 | 300 |
| Electron exposure (e–/Å^2^) | 48 | 48 | 48 | 48 | 48 | 48 | 48 |
| Defocus range (μm) | -1.8 to -0.8 | -1.8 to -0.8 | -1.8 to -0.8 | -1.8 to -0.8 | -1.8 to -0.8 | -1.8 to -0.8 | -1.8 to -0.8 |
| Pixel size (Å) | 0.414 | 0.414 | 0.414 | 0.414 | 0.414 | 0.414 | 0.414 |
| Movies collected | 7,512 | 7,512 | 7,512 | 7,512 | 7,512 | 7,512 | 7,512 |
| Symmetry imposed | C1 | C1 | C1 | C1 | C1 | C1 | C1 |
| FSC threshold | 0.143 | 0.143 | 0.143 | 0.143 | 0.143 | 0.143 | 0.143 |
| Initial particle images (no.) | 746,254 | 746,254 | 746,254 | 746,254 | 746,254 | 746,254 | 746,254 |
| Final particle images (no.) | 132,787 | 109,228 | 186,819 | 70,034 | 40,739 | 22,661 | 26,630 |
| Map resolution (Å) | 2.32 | 2.42 | 2.28 | 2.58 | 2.50 | 2.77 | 2.75 |
| Model resolution (Å) | 2.32 | 1.90 | 2.04 | 2.07 | 2.84 | 2.12 | 2.13 |
| *Model composition* | | | | | | | |
| Non-hydrogen atoms | 17,777 | 17,777 | 17,768 | 17,362 | 16,770 | 16,688 | 14,946 |
| Protein residues | 1449 | 1449 | 1448 | 1398 | 1348 | 1348 | 1226 |
| DNA bases | 306 | 306 | 306 | 306 | 296 | 292 | 256 |
| *R.m.s deviations* | | | | | | | |
| Bond lengths (Å) | 0.005 | 0.006 | 0.005 | 0.005 | 0.004 | 0.005 | 0.005 |
| Bond angles (°) | 0.843 | 0.894 | 0.859 | 0.861 | 0.838 | 0.859 | 0.854 |
| MolProbity score | 1.21 | 1.20 | 1.25 | 1.48 | 1.51 | 1.73 | 1.18 |
| Clashscore | 3.67 | 4.19 | 3.95 | 4.20 | 3.87 | 4.14 | 2.74 |
| Poor rotamers (%) | 1.13 | 0.81 | 1.1776 | 1.51 | 1.74 | 2.17 | 1.06 |
| *Ramachandran plot* | | | | | | | |
| Favored (%) | 97.96 | 98.17 | 97.89 | 97.22 | 97.13 | 95.99 | 97.50 |
| Allowed (%) | 2.04 | 1.76 | 2.11 | 2.78 | 2.87 | 4.01 | 2.50 |
| Outliers (%) | 0 | 0.07 | 0 | 0 | 0 | 0 | 0 |

| **Nucleosome with an SSB at SHL +5.3 bound to PARP2–HPF1 in the presence of talazoparib** | | | | | | | | | |
| --- | --- | --- | --- | --- | --- | --- | --- | --- | --- |
| Class | 1 | 2 | 3 | 4 | 5 | 6 | 7 | 8 | 9 |
| PDB ID | 9ZS5 | 9ZS6 | 9ZSG | 9ZSI | 9ZSJ | 9ZSK | 9ZSL | 9ZSM | 9ZSO |
| EMDB ID | EMD-74691 | EMD-74693 | EMD-74711 | EMD-74712 | EMD-74713 | EMD-74716 | EMD-74717 | EMD-74719 | EMD-74720 |
| *Data collection and processing* | | | | | | | | | |
| Magnification | 105,000 | 105,000 | 105,000 | 105,000 | 105,000 | 105,000 | 105,000 | 105,000 | 105,000 |
| Voltage (kV) | 300 | 300 | 300 | 300 | 300 | 300 | 300 | 300 | 300 |
| Electron exposure (e–/Å^2^) | 49 | 49 | 49 | 49 | 49 | 49 | 49 | 49 | 49 |
| Defocus range (μm) | -1.8 to -0.8 | -1.8 to -0.8 | -1.8 to -0.8 | -1.8 to -0.8 | -1.8 to -0.8 | -1.8 to -0.8 | -1.8 to -0.8 | -1.8 to -0.8 | -1.8 to -0.8 |
| Pixel size (Å) | 0.414 | 0.414 | 0.414 | 0.414 | 0.414 | 0.414 | 0.414 | 0.414 | 0.414 |
| Movies collected | 8,818 | 8,818 | 8,818 | 8,818 | 8,818 | 8,818 | 8,818 | 8,818 | 8,818 |
| Symmetry imposed | C1 | C1 | C1 | C1 | C1 | C1 | C1 | C1 | C1 |
| FSC threshold | 0.143 | 0.143 | 0.143 | 0.143 | 0.143 | 0.143 | 0.143 | 0.143 | 0.143 |
| Initial particle images (no.) | 827,562 | 827,562 | 827,562 | 827,562 | 827,562 | 827,562 | 827,562 | 827,562 | 827,562 |
| Final particle images (no.) | 235,477 | 27,024 | 30,675 | 25,517 | 65,900 | 70,411 | 101,394 | 63,023 | 24,999 |
| Map resolution (Å) | 2.32 | 2.67 | 2.60 | 2.69 | 2.42 | 2.40 | 2.33 | 2.49 | 2.71 |
| Model resolution (Å) | 1.90 | 2.13 | 2.12 | 2.14 | 2.06 | 2.03 | 2.03 | 2.08 | 2.14 |
| *Model composition* | | | | | | | | | |
| Non-hydrogen atoms | 17,564 | 17,564 | 17,565 | 17,165 | 17,564 | 16,966 | 17,355 | 17,197 | 14,946 |
| Protein residues | 1423 | 1423 | 1423 | 1372 | 1423 | 1347 | 1422 | 1418 | 1226 |
| DNA bases | 306 | 306 | 306 | 306 | 306 | 306 | 296 | 290 | 256 |
| *R.m.s deviations* | | | | | | | | | |
| Bond lengths (Å) | 0.005 | 0.005 | 0.005 | 0.004 | 0.004 | 0.005 | 0.004 | 0.005 | 0.005 |
| Bond angles (°) | 0.870 | 0.895 | 0.875 | 0.853 | 0.853 | 0.856 | 0.851 | 0.853 | 0.851 |
| MolProbity score | 1.32 | 1.39 | 1.65 | 1.29 | 1.50 | 1.37 | 1.32 | 1.49 | 1.26 |
| Clashscore | 4.64 | 5.69 | 3.41 | 3.75 | 4.80 | 3.51 | 3.60 | 4.48 | 3.42 |
| Poor rotamers (%) | 0.83 | 0.66 | 2.64 | 1.20 | 1.49 | 1.48 | 1.49 | 1.57 | 0.67 |
| *Ramachandran plot* | | | | | | | | | |
| Favored (%) | 97.63 | 97.63 | 96.84 | 97.69 | 97.35 | 97.50 | 97.85 | 97.41 | 97.34 |
| Allowed (%) | 2.37 | 2.37 | 3.16 | 2.24 | 2.65 | 2.50 | 2.15 | 2.59 | 2.66 |
| Outliers (%) | 0 | 0 | 0 | 0.07 | 0 | 0 | 0 | 0 | 0 |

**Supplementary Table 4. Cryo-EM data collection and model refinement statistics.** Cryo-EM acquisition, reconstruction, and atomic model validation parameters for nucleosome, WGR-only, PARP2 (±HPF1), and PARP2–HPF1 complexes in the absence or presence of PARP inhibitors (olaparib, EB47, talazoparib). For datasets with multiple classes, statistics are reported per class with corresponding PDB and EMDB accession codes. Map resolutions are reported at FSC = 0.143. Model quality metrics include r.m.s. deviations, MolProbity scores, clashscores, rotamer statistics, and Ramachandran analysis.
